# Measurement of jet fragmentation in Pb+Pb and *pp* collisions at $\sqrt{{s_\mathrm{NN}}} = 2.76$ TeV with the ATLAS detector at the LHC

**DOI:** 10.1140/epjc/s10052-017-4915-5

**Published:** 2017-06-08

**Authors:** M. Aaboud, G. Aad, B. Abbott, J. Abdallah, O. Abdinov, B. Abeloos, S. H. Abidi, O. S. AbouZeid, N. L. Abraham, H. Abramowicz, H. Abreu, R. Abreu, Y. Abulaiti, B. S. Acharya, S. Adachi, L. Adamczyk, J. Adelman, M. Adersberger, T. Adye, A. A. Affolder, T. Agatonovic-Jovin, C. Agheorghiesei, J. A. Aguilar-Saavedra, S. P. Ahlen, F. Ahmadov, G. Aielli, S. Akatsuka, H. Akerstedt, T. P. A. Åkesson, A. V. Akimov, G. L. Alberghi, J. Albert, M. J. Alconada Verzini, M. Aleksa, I. N. Aleksandrov, C. Alexa, G. Alexander, T. Alexopoulos, M. Alhroob, B. Ali, M. Aliev, G. Alimonti, J. Alison, S. P. Alkire, B. M. M. Allbrooke, B. W. Allen, P. P. Allport, A. Aloisio, A. Alonso, F. Alonso, C. Alpigiani, A. A. Alshehri, M. Alstaty, B. Alvarez Gonzalez, D. Álvarez Piqueras, M. G. Alviggi, B. T. Amadio, Y. Amaral Coutinho, C. Amelung, D. Amidei, S. P. Amor Dos Santos, A. Amorim, S. Amoroso, G. Amundsen, C. Anastopoulos, L. S. Ancu, N. Andari, T. Andeen, C. F. Anders, J. K. Anders, K. J. Anderson, A. Andreazza, V. Andrei, S. Angelidakis, I. Angelozzi, A. Angerami, F. Anghinolfi, A. V. Anisenkov, N. Anjos, A. Annovi, C. Antel, M. Antonelli, A. Antonov, D. J. Antrim, F. Anulli, M. Aoki, L. Aperio Bella, G. Arabidze, Y. Arai, J. P. Araque, V. Araujo Ferraz, A. T. H. Arce, R. E. Ardell, F. A. Arduh, J-F. Arguin, S. Argyropoulos, M. Arik, A. J. Armbruster, L. J. Armitage, O. Arnaez, H. Arnold, M. Arratia, O. Arslan, A. Artamonov, G. Artoni, S. Artz, S. Asai, N. Asbah, A. Ashkenazi, L. Asquith, K. Assamagan, R. Astalos, M. Atkinson, N. B. Atlay, K. Augsten, G. Avolio, B. Axen, M. K. Ayoub, G. Azuelos, A. E. Baas, M. J. Baca, H. Bachacou, K. Bachas, M. Backes, M. Backhaus, P. Bagiacchi, P. Bagnaia, H. Bahrasemani, J. T. Baines, M. Bajic, O. K. Baker, E. M. Baldin, P. Balek, T. Balestri, F. Balli, W. K. Balunas, E. Banas, Sw. Banerjee, A. A. E. Bannoura, L. Barak, E. L. Barberio, D. Barberis, M. Barbero, T. Barillari, M-S Barisits, T. Barklow, N. Barlow, S. L. Barnes, B. M. Barnett, R. M. Barnett, Z. Barnovska-Blenessy, A. Baroncelli, G. Barone, A. J. Barr, L. Barranco Navarro, F. Barreiro, J. Barreiro Guimarães da Costa, R. Bartoldus, A. E. Barton, P. Bartos, A. Basalaev, A. Bassalat, R. L. Bates, S. J. Batista, J. R. Batley, M. Battaglia, M. Bauce, F. Bauer, H. S. Bawa, J. B. Beacham, M. D. Beattie, T. Beau, P. H. Beauchemin, P. Bechtle, H. P. Beck, K. Becker, M. Becker, M. Beckingham, C. Becot, A. J. Beddall, A. Beddall, V. A. Bednyakov, M. Bedognetti, C. P. Bee, T. A. Beermann, M. Begalli, M. Begel, J. K. Behr, A. S. Bell, G. Bella, L. Bellagamba, A. Bellerive, M. Bellomo, K. Belotskiy, O. Beltramello, N. L. Belyaev, O. Benary, D. Benchekroun, M. Bender, K. Bendtz, N. Benekos, Y. Benhammou, E. Benhar Noccioli, J. Benitez, D. P. Benjamin, M. Benoit, J. R. Bensinger, S. Bentvelsen, L. Beresford, M. Beretta, D. Berge, E. Bergeaas Kuutmann, N. Berger, J. Beringer, S. Berlendis, N. R. Bernard, G. Bernardi, C. Bernius, F. U. Bernlochner, T. Berry, P. Berta, C. Bertella, G. Bertoli, F. Bertolucci, I. A. Bertram, C. Bertsche, D. Bertsche, G. J. Besjes, O. Bessidskaia Bylund, M. Bessner, N. Besson, C. Betancourt, A. Bethani, S. Bethke, A. J. Bevan, R. M. Bianchi, M. Bianco, O. Biebel, D. Biedermann, R. Bielski, N. V. Biesuz, M. Biglietti, J. Bilbao De Mendizabal, T. R. V. Billoud, H. Bilokon, M. Bindi, A. Bingul, C. Bini, S. Biondi, T. Bisanz, C. Bittrich, D. M. Bjergaard, C. W. Black, J. E. Black, K. M. Black, D. Blackburn, R. E. Blair, T. Blazek, I. Bloch, C. Blocker, A. Blue, W. Blum, U. Blumenschein, S. Blunier, G. J. Bobbink, V. S. Bobrovnikov, S. S. Bocchetta, A. Bocci, C. Bock, M. Boehler, D. Boerner, D. Bogavac, A. G. Bogdanchikov, C. Bohm, V. Boisvert, P. Bokan, T. Bold, A. S. Boldyrev, M. Bomben, M. Bona, M. Boonekamp, A. Borisov, G. Borissov, J. Bortfeldt, D. Bortoletto, V. Bortolotto, K. Bos, D. Boscherini, M. Bosman, J. D. Bossio Sola, J. Boudreau, J. Bouffard, E. V. Bouhova-Thacker, D. Boumediene, C. Bourdarios, S. K. Boutle, A. Boveia, J. Boyd, I. R. Boyko, J. Bracinik, A. Brandt, G. Brandt, O. Brandt, U. Bratzler, B. Brau, J. E. Brau, W. D. Breaden Madden, K. Brendlinger, A. J. Brennan, L. Brenner, R. Brenner, S. Bressler, D. L. Briglin, T. M. Bristow, D. Britton, D. Britzger, F. M. Brochu, I. Brock, R. Brock, G. Brooijmans, T. Brooks, W. K. Brooks, J. Brosamer, E. Brost, J. H Broughton, P. A. Bruckman de Renstrom, D. Bruncko, A. Bruni, G. Bruni, L. S. Bruni, BH Brunt, M. Bruschi, N. Bruscino, P. Bryant, L. Bryngemark, T. Buanes, Q. Buat, P. Buchholz, A. G. Buckley, I. A. Budagov, F. Buehrer, M. K. Bugge, O. Bulekov, D. Bullock, H. Burckhart, S. Burdin, C. D. Burgard, A. M. Burger, B. Burghgrave, K. Burka, S. Burke, I. Burmeister, J. T. P. Burr, E. Busato, D. Büscher, V. Büscher, P. Bussey, J. M. Butler, C. M. Buttar, J. M. Butterworth, P. Butti, W. Buttinger, A. Buzatu, A. R. Buzykaev, S. Cabrera Urbán, D. Caforio, V. M. Cairo, O. Cakir, N. Calace, P. Calafiura, A. Calandri, G. Calderini, P. Calfayan, G. Callea, L. P. Caloba, S. Calvente Lopez, D. Calvet, S. Calvet, T. P. Calvet, R. Camacho Toro, S. Camarda, P. Camarri, D. Cameron, R. Caminal Armadans, C. Camincher, S. Campana, M. Campanelli, A. Camplani, A. Campoverde, V. Canale, M. Cano Bret, J. Cantero, T. Cao, M. D. M. Capeans Garrido, I. Caprini, M. Caprini, M. Capua, R. M. Carbone, R. Cardarelli, F. Cardillo, I. Carli, T. Carli, G. Carlino, B. T. Carlson, L. Carminati, R. M. D. Carney, S. Caron, E. Carquin, G. D. Carrillo-Montoya, J. Carvalho, D. Casadei, M. P. Casado, M. Casolino, D. W. Casper, R. Castelijn, A. Castelli, V. Castillo Gimenez, N. F. Castro, A. Catinaccio, J. R. Catmore, A. Cattai, J. Caudron, V. Cavaliere, E. Cavallaro, D. Cavalli, M. Cavalli-Sforza, V. Cavasinni, E. Celebi, F. Ceradini, L. Cerda Alberich, A. S. Cerqueira, A. Cerri, L. Cerrito, F. Cerutti, A. Cervelli, S. A. Cetin, A. Chafaq, D. Chakraborty, S. K. Chan, W. S. Chan, Y. L. Chan, P. Chang, J. D. Chapman, D. G. Charlton, A. Chatterjee, C. C. Chau, C. A. Chavez Barajas, S. Che, S. Cheatham, A. Chegwidden, S. Chekanov, S. V. Chekulaev, G. A. Chelkov, M. A. Chelstowska, C. Chen, H. Chen, S. Chen, S. Chen, X. Chen, Y. Chen, H. C. Cheng, H. J. Cheng, Y. Cheng, A. Cheplakov, E. Cheremushkina, R. Cherkaoui El Moursli, V. Chernyatin, E. Cheu, L. Chevalier, V. Chiarella, G. Chiarelli, G. Chiodini, A. S. Chisholm, A. Chitan, Y. H. Chiu, M. V. Chizhov, K. Choi, A. R. Chomont, S. Chouridou, B. K. B. Chow, V. Christodoulou, D. Chromek-Burckhart, M. C. Chu, J. Chudoba, A. J. Chuinard, J. J. Chwastowski, L. Chytka, A. K. Ciftci, D. Cinca, V. Cindro, I. A. Cioara, C. Ciocca, A. Ciocio, F. Cirotto, Z. H. Citron, M. Citterio, M. Ciubancan, A. Clark, B. L. Clark, M. R. Clark, P. J. Clark, R. N. Clarke, C. Clement, Y. Coadou, M. Cobal, A. Coccaro, J. Cochran, L. Colasurdo, B. Cole, A. P. Colijn, J. Collot, T. Colombo, P. Conde Muiño, E. Coniavitis, S. H. Connell, I. A. Connelly, V. Consorti, S. Constantinescu, G. Conti, F. Conventi, M. Cooke, B. D. Cooper, A. M. Cooper-Sarkar, F. Cormier, K. J. R. Cormier, M. Corradi, F. Corriveau, A. Cortes-Gonzalez, G. Cortiana, G. Costa, M. J. Costa, D. Costanzo, G. Cottin, G. Cowan, B. E. Cox, K. Cranmer, S. J. Crawley, R. A. Creager, G. Cree, S. Crépé-Renaudin, F. Crescioli, W. A. Cribbs, M. Crispin Ortuzar, M. Cristinziani, V. Croft, G. Crosetti, A. Cueto, T. Cuhadar Donszelmann, A. R. Cukierman, J. Cummings, M. Curatolo, J. Cúth, H. Czirr, P. Czodrowski, G. D’amen, S. D’Auria, M. D’Onofrio, M. J. Da Cunha Sargedas De Sousa, C. Da Via, W. Dabrowski, T. Dado, T. Dai, O. Dale, F. Dallaire, C. Dallapiccola, M. Dam, J. R. Dandoy, N. P. Dang, A. C. Daniells, N. S. Dann, M. Danninger, M. Dano Hoffmann, V. Dao, G. Darbo, S. Darmora, J. Dassoulas, A. Dattagupta, T. Daubney, W. Davey, C. David, T. Davidek, M. Davies, P. Davison, E. Dawe, I. Dawson, K. De, R. de Asmundis, A. De Benedetti, S. De Castro, S. De Cecco, N. De Groot, P. de Jong, H. De la Torre, F. De Lorenzi, A. De Maria, D. De Pedis, A. De Salvo, U. De Sanctis, A. De Santo, K. De Vasconcelos Corga, J. B. De Vivie De Regie, W. J. Dearnaley, R. Debbe, C. Debenedetti, D. V. Dedovich, N. Dehghanian, I. Deigaard, M. Del Gaudio, J. Del Peso, T. Del Prete, D. Delgove, F. Deliot, C. M. Delitzsch, A. Dell’Acqua, L. Dell’Asta, M. Dell’Orso, M. Della Pietra, D. della Volpe, M. Delmastro, C. Delporte, P. A. Delsart, D. A. DeMarco, S. Demers, M. Demichev, A. Demilly, S. P. Denisov, D. Denysiuk, D. Derendarz, J. E. Derkaoui, F. Derue, P. Dervan, K. Desch, C. Deterre, K. Dette, P. O. Deviveiros, A. Dewhurst, S. Dhaliwal, A. Di Ciaccio, L. Di Ciaccio, W. K. Di Clemente, C. Di Donato, A. Di Girolamo, B. Di Girolamo, B. Di Micco, R. Di Nardo, K. F. Di Petrillo, A. Di Simone, R. Di Sipio, D. Di Valentino, C. Diaconu, M. Diamond, F. A. Dias, M. A. Diaz, E. B. Diehl, J. Dietrich, S. Díez Cornell, A. Dimitrievska, J. Dingfelder, P. Dita, S. Dita, F. Dittus, F. Djama, T. Djobava, J. I. Djuvsland, M. A. B. do Vale, D. Dobos, M. Dobre, C. Doglioni, J. Dolejsi, Z. Dolezal, M. Donadelli, S. Donati, P. Dondero, J. Donini, J. Dopke, A. Doria, M. T. Dova, A. T. Doyle, E. Drechsler, M. Dris, Y. Du, J. Duarte-Campderros, E. Duchovni, G. Duckeck, A. Ducourthial, O. A. Ducu, D. Duda, A. Dudarev, A. C. Dudder, E. M. Duffield, L. Duflot, M. Dührssen, M. Dumancic, A. E. Dumitriu, A. K. Duncan, M. Dunford, H. Duran Yildiz, M. Düren, A. Durglishvili, D. Duschinger, B. Dutta, M. Dyndal, C. Eckardt, K. M. Ecker, R. C. Edgar, T. Eifert, G. Eigen, K. Einsweiler, T. Ekelof, M. El Kacimi, R. El Kosseifi, V. Ellajosyula, M. Ellert, S. Elles, F. Ellinghaus, A. A. Elliot, N. Ellis, J. Elmsheuser, M. Elsing, D. Emeliyanov, Y. Enari, O. C. Endner, J. S. Ennis, J. Erdmann, A. Ereditato, G. Ernis, M. Ernst, S. Errede, E. Ertel, M. Escalier, H. Esch, C. Escobar, B. Esposito, O. Estrada Pastor, A. I. Etienvre, E. Etzion, H. Evans, A. Ezhilov, M. Ezzi, F. Fabbri, L. Fabbri, G. Facini, R. M. Fakhrutdinov, S. Falciano, R. J. Falla, J. Faltova, Y. Fang, M. Fanti, A. Farbin, A. Farilla, C. Farina, E. M. Farina, T. Farooque, S. Farrell, S. M. Farrington, P. Farthouat, F. Fassi, P. Fassnacht, D. Fassouliotis, M. Faucci Giannelli, A. Favareto, W. J. Fawcett, L. Fayard, O. L. Fedin, W. Fedorko, S. Feigl, L. Feligioni, C. Feng, E. J. Feng, H. Feng, A. B. Fenyuk, L. Feremenga, P. Fernandez Martinez, S. Fernandez Perez, J. Ferrando, A. Ferrari, P. Ferrari, R. Ferrari, D. E. Ferreira de Lima, A. Ferrer, D. Ferrere, C. Ferretti, F. Fiedler, A. Filipčič, M. Filipuzzi, F. Filthaut, M. Fincke-Keeler, K. D. Finelli, M. C. N. Fiolhais, L. Fiorini, A. Fischer, C. Fischer, J. Fischer, W. C. Fisher, N. Flaschel, I. Fleck, P. Fleischmann, R. R. M. Fletcher, T. Flick, B. M. Flierl, L. R. Flores Castillo, M. J. Flowerdew, F. A. Foerster, G. T. Forcolin, A. Formica, A. Forti, A. G. Foster, D. Fournier, H. Fox, S. Fracchia, P. Francavilla, M. Franchini, S. Franchino, D. Francis, L. Franconi, M. Franklin, M. Frate, M. Fraternali, D. Freeborn, S. M. Fressard-Batraneanu, B. Freund, D. Froidevaux, J. A. Frost, C. Fukunaga, E. Fullana Torregrosa, T. Fusayasu, J. Fuster, C. Gabaldon, O. Gabizon, A. Gabrielli, A. Gabrielli, G. P. Gach, S. Gadatsch, S. Gadomski, G. Gagliardi, L. G. Gagnon, P. Gagnon, C. Galea, B. Galhardo, E. J. Gallas, B. J. Gallop, P. Gallus, G. Galster, K. K. Gan, S. Ganguly, J. Gao, Y. Gao, Y. S. Gao, F. M. Garay Walls, C. García, J. E. García Navarro, M. Garcia-Sciveres, R. W. Gardner, N. Garelli, V. Garonne, A. Gascon Bravo, K. Gasnikova, C. Gatti, A. Gaudiello, G. Gaudio, I. L. Gavrilenko, C. Gay, G. Gaycken, E. N. Gazis, C. N. P. Gee, M. Geisen, M. P. Geisler, K. Gellerstedt, C. Gemme, M. H. Genest, C. Geng, S. Gentile, C. Gentsos, S. George, D. Gerbaudo, A. Gershon, S. Ghasemi, M. Ghneimat, B. Giacobbe, S. Giagu, P. Giannetti, S. M. Gibson, M. Gignac, M. Gilchriese, D. Gillberg, G. Gilles, D. M. Gingrich, N. Giokaris, M. P. Giordani, F. M. Giorgi, P. F. Giraud, P. Giromini, D. Giugni, F. Giuli, C. Giuliani, M. Giulini, B. K. Gjelsten, S. Gkaitatzis, I. Gkialas, E. L. Gkougkousis, L. K. Gladilin, C. Glasman, J. Glatzer, P. C. F. Glaysher, A. Glazov, M. Goblirsch-Kolb, J. Godlewski, S. Goldfarb, T. Golling, D. Golubkov, A. Gomes, R. Gonçalo, R. Goncalves Gama, J. Goncalves Pinto Firmino Da Costa, G. Gonella, L. Gonella, A. Gongadze, S. González de la Hoz, S. Gonzalez-Sevilla, L. Goossens, P. A. Gorbounov, H. A. Gordon, I. Gorelov, B. Gorini, E. Gorini, A. Gorišek, A. T. Goshaw, C. Gössling, M. I. Gostkin, C. R. Goudet, D. Goujdami, A. G. Goussiou, N. Govender, E. Gozani, L. Graber, I. Grabowska-Bold, P. O. J. Gradin, J. Gramling, E. Gramstad, S. Grancagnolo, V. Gratchev, P. M. Gravila, C. Gray, H. M. Gray, Z. D. Greenwood, C. Grefe, K. Gregersen, I. M. Gregor, P. Grenier, K. Grevtsov, J. Griffiths, A. A. Grillo, K. Grimm, S. Grinstein, Ph. Gris, J. -F. Grivaz, S. Groh, E. Gross, J. Grosse-Knetter, G. C. Grossi, Z. J. Grout, A. Grummer, L. Guan, W. Guan, J. Guenther, F. Guescini, D. Guest, O. Gueta, B. Gui, E. Guido, T. Guillemin, S. Guindon, U. Gul, C. Gumpert, J. Guo, W. Guo, Y. Guo, R. Gupta, S. Gupta, G. Gustavino, P. Gutierrez, N. G. Gutierrez Ortiz, C. Gutschow, C. Guyot, M. P. Guzik, C. Gwenlan, C. B. Gwilliam, A. Haas, C. Haber, H. K. Hadavand, N. Haddad, A. Hadef, S. Hageböck, M. Hagihara, H. Hakobyan, M. Haleem, J. Haley, G. Halladjian, G. D. Hallewell, K. Hamacher, P. Hamal, K. Hamano, A. Hamilton, G. N. Hamity, P. G. Hamnett, L. Han, S. Han, K. Hanagaki, K. Hanawa, M. Hance, B. Haney, P. Hanke, J. B. Hansen, J. D. Hansen, M. C. Hansen, P. H. Hansen, K. Hara, A. S. Hard, T. Harenberg, F. Hariri, S. Harkusha, R. D. Harrington, P. F. Harrison, F. Hartjes, N. M. Hartmann, M. Hasegawa, Y. Hasegawa, A. Hasib, S. Hassani, S. Haug, R. Hauser, L. Hauswald, L. B. Havener, M. Havranek, C. M. Hawkes, R. J. Hawkings, D. Hayakawa, D. Hayden, C. P. Hays, J. M. Hays, H. S. Hayward, S. J. Haywood, S. J. Head, T. Heck, V. Hedberg, L. Heelan, K. K. Heidegger, S. Heim, T. Heim, B. Heinemann, J. J. Heinrich, L. Heinrich, C. Heinz, J. Hejbal, L. Helary, A. Held, S. Hellman, C. Helsens, J. Henderson, R. C. W. Henderson, Y. Heng, S. Henkelmann, A. M. Henriques Correia, S. Henrot-Versille, G. H. Herbert, H. Herde, V. Herget, Y. Hernández Jiménez, G. Herten, R. Hertenberger, L. Hervas, T. C. Herwig, G. G. Hesketh, N. P. Hessey, J. W. Hetherly, S. Higashino, E. Higón-Rodriguez, E. Hill, J. C. Hill, K. H. Hiller, S. J. Hillier, I. Hinchliffe, M. Hirose, D. Hirschbuehl, B. Hiti, O. Hladik, X. Hoad, J. Hobbs, N. Hod, M. C. Hodgkinson, P. Hodgson, A. Hoecker, M. R. Hoeferkamp, F. Hoenig, D. Hohn, T. R. Holmes, M. Homann, S. Honda, T. Honda, T. M. Hong, B. H. Hooberman, W. H. Hopkins, Y. Horii, A. J. Horton, J-Y. Hostachy, S. Hou, A. Hoummada, J. Howarth, J. Hoya, M. Hrabovsky, I. Hristova, J. Hrivnac, T. Hryn’ova, A. Hrynevich, P. J. Hsu, S.-C. Hsu, Q. Hu, S. Hu, Y. Huang, Z. Hubacek, F. Hubaut, F. Huegging, T. B. Huffman, E. W. Hughes, G. Hughes, M. Huhtinen, P. Huo, N. Huseynov, J. Huston, J. Huth, G. Iacobucci, G. Iakovidis, I. Ibragimov, L. Iconomidou-Fayard, Z. Idrissi, P. Iengo, O. Igonkina, T. Iizawa, Y. Ikegami, M. Ikeno, Y. Ilchenko, D. Iliadis, N. Ilic, G. Introzzi, P. Ioannou, M. Iodice, K. Iordanidou, V. Ippolito, N. Ishijima, M. Ishino, M. Ishitsuka, C. Issever, S. Istin, F. Ito, J. M. Iturbe Ponce, R. Iuppa, H. Iwasaki, J. M. Izen, V. Izzo, S. Jabbar, P. Jackson, V. Jain, K. B. Jakobi, K. Jakobs, S. Jakobsen, T. Jakoubek, D. O. Jamin, D. K. Jana, R. Jansky, J. Janssen, M. Janus, P. A. Janus, G. Jarlskog, N. Javadov, T. Javůrek, M. Javurkova, F. Jeanneau, L. Jeanty, J. Jejelava, A. Jelinskas, P. Jenni, C. Jeske, S. Jézéquel, H. Ji, J. Jia, H. Jiang, Y. Jiang, Z. Jiang, S. Jiggins, J. Jimenez Pena, S. Jin, A. Jinaru, O. Jinnouchi, H. Jivan, P. Johansson, K. A. Johns, C. A. Johnson, W. J. Johnson, K. Jon-And, R. W. L. Jones, S. D. Jones, S. Jones, T. J. Jones, J. Jongmanns, P. M. Jorge, J. Jovicevic, X. Ju, A. Juste Rozas, M. K. Köhler, A. Kaczmarska, M. Kado, H. Kagan, M. Kagan, S. J. Kahn, T. Kaji, E. Kajomovitz, C. W. Kalderon, A. Kaluza, S. Kama, A. Kamenshchikov, N. Kanaya, S. Kaneti, L. Kanjir, V. A. Kantserov, J. Kanzaki, B. Kaplan, L. S. Kaplan, D. Kar, K. Karakostas, N. Karastathis, M. J. Kareem, E. Karentzos, S. N. Karpov, Z. M. Karpova, K. Karthik, V. Kartvelishvili, A. N. Karyukhin, K. Kasahara, L. Kashif, R. D. Kass, A. Kastanas, Y. Kataoka, C. Kato, A. Katre, J. Katzy, K. Kawade, K. Kawagoe, T. Kawamoto, G. Kawamura, E. F. Kay, V. F. Kazanin, R. Keeler, R. Kehoe, J. S. Keller, J. J. Kempster, H. Keoshkerian, O. Kepka, B. P. Kerševan, S. Kersten, R. A. Keyes, M. Khader, F. Khalil-zada, A. Khanov, A. G. Kharlamov, T. Kharlamova, A. Khodinov, T. J. Khoo, V. Khovanskiy, E. Khramov, J. Khubua, S. Kido, C. R. Kilby, H. Y. Kim, S. H. Kim, Y. K. Kim, N. Kimura, O. M. Kind, B. T. King, D. Kirchmeier, J. Kirk, A. E. Kiryunin, T. Kishimoto, D. Kisielewska, K. Kiuchi, O. Kivernyk, E. Kladiva, T. Klapdor-Kleingrothaus, M. H. Klein, M. Klein, U. Klein, K. Kleinknecht, P. Klimek, A. Klimentov, R. Klingenberg, T. Klingl, T. Klioutchnikova, E. -E. Kluge, P. Kluit, S. Kluth, J. Knapik, E. Kneringer, E. B. F. G. Knoops, A. Knue, A. Kobayashi, D. Kobayashi, T. Kobayashi, M. Kobel, M. Kocian, P. Kodys, T. Koffas, E. Koffeman, N. M. Köhler, T. Koi, M. Kolb, I. Koletsou, A. A. Komar, Y. Komori, T. Kondo, N. Kondrashova, K. Köneke, A. C. König, T. Kono, R. Konoplich, N. Konstantinidis, R. Kopeliansky, S. Koperny, A. K. Kopp, K. Korcyl, K. Kordas, A. Korn, A. A. Korol, I. Korolkov, E. V. Korolkova, O. Kortner, S. Kortner, T. Kosek, V. V. Kostyukhin, A. Kotwal, A. Koulouris, A. Kourkoumeli-Charalampidi, C. Kourkoumelis, E. Kourlitis, V. Kouskoura, A. B. Kowalewska, R. Kowalewski, T. Z. Kowalski, C. Kozakai, W. Kozanecki, A. S. Kozhin, V. A. Kramarenko, G. Kramberger, D. Krasnopevtsev, M. W. Krasny, A. Krasznahorkay, D. Krauss, A. Kravchenko, J. A. Kremer, M. Kretz, J. Kretzschmar, K. Kreutzfeldt, P. Krieger, K. Krizka, K. Kroeninger, H. Kroha, J. Kroll, J. Kroll, J. Kroseberg, J. Krstic, U. Kruchonak, H. Krüger, N. Krumnack, M. C. Kruse, M. Kruskal, T. Kubota, H. Kucuk, S. Kuday, J. T. Kuechler, S. Kuehn, A. Kugel, F. Kuger, T. Kuhl, V. Kukhtin, R. Kukla, Y. Kulchitsky, S. Kuleshov, Y. P. Kulinich, M. Kuna, T. Kunigo, A. Kupco, O. Kuprash, H. Kurashige, L. L. Kurchaninov, Y. A. Kurochkin, M. G. Kurth, V. Kus, E. S. Kuwertz, M. Kuze, J. Kvita, T. Kwan, D. Kyriazopoulos, A. La Rosa, J. L. La Rosa Navarro, L. La Rotonda, C. Lacasta, F. Lacava, J. Lacey, H. Lacker, D. Lacour, E. Ladygin, R. Lafaye, B. Laforge, T. Lagouri, S. Lai, S. Lammers, W. Lampl, E. Lançon, U. Landgraf, M. P. J. Landon, M. C. Lanfermann, V. S. Lang, J. C. Lange, A. J. Lankford, F. Lanni, K. Lantzsch, A. Lanza, A. Lapertosa, S. Laplace, J. F. Laporte, T. Lari, F. Lasagni Manghi, M. Lassnig, P. Laurelli, W. Lavrijsen, A. T. Law, P. Laycock, T. Lazovich, M. Lazzaroni, B. Le, O. Le Dortz, E. Le Guirriec, E. P. Le Quilleuc, M. LeBlanc, T. LeCompte, F. Ledroit-Guillon, C. A. Lee, G. R. Lee, S. C. Lee, L. Lee, B. Lefebvre, G. Lefebvre, M. Lefebvre, F. Legger, C. Leggett, A. Lehan, G. Lehmann Miotto, X. Lei, W. A. Leight, M. A. L. Leite, R. Leitner, D. Lellouch, B. Lemmer, K. J. C. Leney, T. Lenz, B. Lenzi, R. Leone, S. Leone, C. Leonidopoulos, G. Lerner, C. Leroy, A. A. J. Lesage, C. G. Lester, M. Levchenko, J. Levêque, D. Levin, L. J. Levinson, M. Levy, D. Lewis, M. Leyton, B. Li, C. Li, H. Li, L. Li, Q. Li, S. Li, X. Li, Y. Li, Z. Liang, B. Liberti, A. Liblong, K. Lie, J. Liebal, W. Liebig, A. Limosani, S. C. Lin, T. H. Lin, B. E. Lindquist, A. E. Lionti, E. Lipeles, A. Lipniacka, M. Lisovyi, T. M. Liss, A. Lister, A. M. Litke, B. Liu, H. Liu, H. Liu, J. K. K. Liu, J. Liu, J. B. Liu, K. Liu, L. Liu, M. Liu, Y. L. Liu, Y. Liu, M. Livan, A. Lleres, J. Llorente Merino, S. L. Lloyd, C. Y. Lo, F. Lo Sterzo, E. M. Lobodzinska, P. Loch, F. K. Loebinger, K. M. Loew, A. Loginov, T. Lohse, K. Lohwasser, M. Lokajicek, B. A. Long, J. D. Long, R. E. Long, L. Longo, K. A. Looper, J. A. Lopez, D. Lopez Mateos, I. Lopez Paz, A. Lopez Solis, J. Lorenz, N. Lorenzo Martinez, M. Losada, P. J. Lösel, X. Lou, A. Lounis, J. Love, P. A. Love, H. Lu, N. Lu, Y. J. Lu, H. J. Lubatti, C. Luci, A. Lucotte, C. Luedtke, F. Luehring, W. Lukas, L. Luminari, O. Lundberg, B. Lund-Jensen, P. M. Luzi, D. Lynn, R. Lysak, E. Lytken, V. Lyubushkin, H. Ma, L. L. Ma, Y. Ma, G. Maccarrone, A. Macchiolo, C. M. Macdonald, B. Maček, J. Machado Miguens, D. Madaffari, R. Madar, H. J. Maddocks, W. F. Mader, A. Madsen, J. Maeda, S. Maeland, T. Maeno, A. Maevskiy, E. Magradze, J. Mahlstedt, C. Maiani, C. Maidantchik, A. A. Maier, T. Maier, A. Maio, S. Majewski, Y. Makida, N. Makovec, B. Malaescu, Pa. Malecki, V. P. Maleev, F. Malek, U. Mallik, D. Malon, C. Malone, S. Maltezos, S. Malyukov, J. Mamuzic, G. Mancini, L. Mandelli, I. Mandić, J. Maneira, L. Manhaes de Andrade Filho, J. Manjarres Ramos, A. Mann, A. Manousos, B. Mansoulie, J. D. Mansour, R. Mantifel, M. Mantoani, S. Manzoni, L. Mapelli, G. Marceca, L. March, L. Marchese, G. Marchiori, M. Marcisovsky, M. Marjanovic, D. E. Marley, F. Marroquim, S. P. Marsden, Z. Marshall, M. U. F Martensson, S. Marti-Garcia, C. B. Martin, T. A. Martin, V. J. Martin, B. Martin dit Latour, M. Martinez, V. I. Martinez Outschoorn, S. Martin-Haugh, V. S. Martoiu, A. C. Martyniuk, A. Marzin, L. Masetti, T. Mashimo, R. Mashinistov, J. Masik, A. L. Maslennikov, L. Massa, P. Mastrandrea, A. Mastroberardino, T. Masubuchi, P. Mättig, J. Maurer, S. J. Maxfield, D. A. Maximov, R. Mazini, I. Maznas, S. M. Mazza, N. C. Mc Fadden, G. Mc Goldrick, S. P. Mc Kee, A. McCarn, R. L. McCarthy, T. G. McCarthy, L. I. McClymont, E. F. McDonald, J. A. Mcfayden, G. Mchedlidze, S. J. McMahon, P. C. McNamara, R. A. McPherson, S. Meehan, T. J. Megy, S. Mehlhase, A. Mehta, T. Meideck, K. Meier, C. Meineck, B. Meirose, D. Melini, B. R. Mellado Garcia, M. Melo, F. Meloni, S. B. Menary, L. Meng, X. T. Meng, A. Mengarelli, S. Menke, E. Meoni, S. Mergelmeyer, P. Mermod, L. Merola, C. Meroni, F. S. Merritt, A. Messina, J. Metcalfe, A. S. Mete, C. Meyer, J-P. Meyer, J. Meyer, H. Meyer Zu Theenhausen, F. Miano, R. P. Middleton, S. Miglioranzi, L. Mijović, G. Mikenberg, M. Mikestikova, M. Mikuž, M. Milesi, A. Milic, D. W. Miller, C. Mills, A. Milov, D. A. Milstead, A. A. Minaenko, Y. Minami, I. A. Minashvili, A. I. Mincer, B. Mindur, M. Mineev, Y. Minegishi, Y. Ming, L. M. Mir, K. P. Mistry, T. Mitani, J. Mitrevski, V. A. Mitsou, A. Miucci, P. S. Miyagawa, A. Mizukami, J. U. Mjörnmark, M. Mlynarikova, T. Moa, K. Mochizuki, P. Mogg, S. Mohapatra, S. Molander, R. Moles-Valls, R. Monden, M. C. Mondragon, K. Mönig, J. Monk, E. Monnier, A. Montalbano, J. Montejo Berlingen, F. Monticelli, S. Monzani, R. W. Moore, N. Morange, D. Moreno, M. Moreno Llácer, P. Morettini, S. Morgenstern, D. Mori, T. Mori, M. Morii, M. Morinaga, V. Morisbak, A. K. Morley, G. Mornacchi, J. D. Morris, L. Morvaj, P. Moschovakos, M. Mosidze, H. J. Moss, J. Moss, K. Motohashi, R. Mount, E. Mountricha, E. J. W. Moyse, S. Muanza, R. D. Mudd, F. Mueller, J. Mueller, R. S. P. Mueller, D. Muenstermann, P. Mullen, G. A. Mullier, F. J. Munoz Sanchez, W. J. Murray, H. Musheghyan, M. Muškinja, A. G. Myagkov, M. Myska, B. P. Nachman, O. Nackenhorst, K. Nagai, R. Nagai, K. Nagano, Y. Nagasaka, K. Nagata, M. Nagel, E. Nagy, A. M. Nairz, Y. Nakahama, K. Nakamura, T. Nakamura, I. Nakano, R. F. Naranjo Garcia, R. Narayan, D. I. Narrias Villar, I. Naryshkin, T. Naumann, G. Navarro, R. Nayyar, H. A. Neal, P. Yu. Nechaeva, T. J. Neep, A. Negri, M. Negrini, S. Nektarijevic, C. Nellist, A. Nelson, M. E. Nelson, S. Nemecek, P. Nemethy, A. A. Nepomuceno, M. Nessi, M. S. Neubauer, M. Neumann, P. R. Newman, T. Y. Ng, T. Nguyen Manh, R. B. Nickerson, R. Nicolaidou, J. Nielsen, V. Nikolaenko, I. Nikolic-Audit, K. Nikolopoulos, J. K. Nilsen, P. Nilsson, Y. Ninomiya, A. Nisati, N. Nishu, R. Nisius, T. Nobe, Y. Noguchi, M. Nomachi, I. Nomidis, M. A. Nomura, T. Nooney, M. Nordberg, N. Norjoharuddeen, O. Novgorodova, S. Nowak, M. Nozaki, L. Nozka, K. Ntekas, E. Nurse, F. Nuti, K. O’connor, D. C. O’Neil, A. A. O’Rourke, V. O’Shea, F. G. Oakham, H. Oberlack, T. Obermann, J. Ocariz, A. Ochi, I. Ochoa, J. P. Ochoa-Ricoux, S. Oda, S. Odaka, H. Ogren, A. Oh, S. H. Oh, C. C. Ohm, H. Ohman, H. Oide, H. Okawa, Y. Okumura, T. Okuyama, A. Olariu, L. F. Oleiro Seabra, S. A. Olivares Pino, D. Oliveira Damazio, A. Olszewski, J. Olszowska, A. Onofre, K. Onogi, P. U. E. Onyisi, M. J. Oreglia, Y. Oren, D. Orestano, N. Orlando, R. S. Orr, B. Osculati, R. Ospanov, G. Otero y Garzon, H. Otono, M. Ouchrif, F. Ould-Saada, A. Ouraou, K. P. Oussoren, Q. Ouyang, M. Owen, R. E. Owen, V. E. Ozcan, N. Ozturk, K. Pachal, A. Pacheco Pages, L. Pacheco Rodriguez, C. Padilla Aranda, S. Pagan Griso, M. Paganini, F. Paige, P. Pais, G. Palacino, S. Palazzo, S. Palestini, M. Palka, D. Pallin, E. St. Panagiotopoulou, I. Panagoulias, C. E. Pandini, J. G. Panduro Vazquez, P. Pani, S. Panitkin, D. Pantea, L. Paolozzi, Th. D. Papadopoulou, K. Papageorgiou, A. Paramonov, D. Paredes Hernandez, A. J. Parker, M. A. Parker, K. A. Parker, F. Parodi, J. A. Parsons, U. Parzefall, V. R. Pascuzzi, J. M. Pasner, E. Pasqualucci, S. Passaggio, Fr. Pastore, S. Pataraia, J. R. Pater, T. Pauly, J. Pearce, B. Pearson, S. Pedraza Lopez, R. Pedro, S. V. Peleganchuk, O. Penc, C. Peng, H. Peng, J. Penwell, B. S. Peralva, M. M. Perego, D. V. Perepelitsa, L. Perini, H. Pernegger, S. Perrella, R. Peschke, V. D. Peshekhonov, K. Peters, R. F. Y. Peters, B. A. Petersen, T. C. Petersen, E. Petit, A. Petridis, C. Petridou, P. Petroff, E. Petrolo, M. Petrov, F. Petrucci, N. E. Pettersson, A. Peyaud, R. Pezoa, P. W. Phillips, G. Piacquadio, E. Pianori, A. Picazio, E. Piccaro, M. A. Pickering, R. Piegaia, J. E. Pilcher, A. D. Pilkington, A. W. J. Pin, M. Pinamonti, J. L. Pinfold, H. Pirumov, M. Pitt, L. Plazak, M. -A. Pleier, V. Pleskot, E. Plotnikova, D. Pluth, P. Podberezko, R. Poettgen, R. Poggi, L. Poggioli, D. Pohl, G. Polesello, A. Poley, A. Policicchio, R. Polifka, A. Polini, C. S. Pollard, V. Polychronakos, K. Pommès, D. Ponomarenko, L. Pontecorvo, B. G. Pope, G. A. Popeneciu, A. Poppleton, S. Pospisil, K. Potamianos, I. N. Potrap, C. J. Potter, C. T. Potter, G. Poulard, J. Poveda, M. E. Pozo Astigarraga, P. Pralavorio, A. Pranko, S. Prell, D. Price, L. E. Price, M. Primavera, S. Prince, N. Proklova, K. Prokofiev, F. Prokoshin, S. Protopopescu, J. Proudfoot, M. Przybycien, D. Puddu, A. Puri, P. Puzo, J. Qian, G. Qin, Y. Qin, A. Quadt, W. B. Quayle, M. Queitsch-Maitland, D. Quilty, S. Raddum, V. Radeka, V. Radescu, S. K. Radhakrishnan, P. Radloff, P. Rados, F. Ragusa, G. Rahal, J. A. Raine, S. Rajagopalan, C. Rangel-Smith, M. G. Ratti, D. M. Rauch, F. Rauscher, S. Rave, T. Ravenscroft, I. Ravinovich, J. H. Rawling, M. Raymond, A. L. Read, N. P. Readioff, M. Reale, D. M. Rebuzzi, A. Redelbach, G. Redlinger, R. Reece, R. G. Reed, K. Reeves, L. Rehnisch, J. Reichert, A. Reiss, C. Rembser, H. Ren, M. Rescigno, S. Resconi, E. D. Resseguie, S. Rettie, E. Reynolds, O. L. Rezanova, P. Reznicek, R. Rezvani, R. Richter, S. Richter, E. Richter-Was, O. Ricken, M. Ridel, P. Rieck, C. J. Riegel, J. Rieger, O. Rifki, M. Rijssenbeek, A. Rimoldi, M. Rimoldi, L. Rinaldi, B. Ristić, E. Ritsch, I. Riu, F. Rizatdinova, E. Rizvi, C. Rizzi, R. T. Roberts, S. H. Robertson, A. Robichaud-Veronneau, D. Robinson, J. E. M. Robinson, A. Robson, E. Rocco, C. Roda, Y. Rodina, S. Rodriguez Bosca, A. Rodriguez Perez, D. Rodriguez Rodriguez, S. Roe, C. S. Rogan, O. Røhne, J. Roloff, A. Romaniouk, M. Romano, S. M. Romano Saez, E. Romero Adam, N. Rompotis, M. Ronzani, L. Roos, S. Rosati, K. Rosbach, P. Rose, N.-A. Rosien, V. Rossetti, E. Rossi, L. P. Rossi, J. H. N. Rosten, R. Rosten, M. Rotaru, I. Roth, J. Rothberg, D. Rousseau, A. Rozanov, Y. Rozen, X. Ruan, F. Rubbo, F. Rühr, A. Ruiz-Martinez, Z. Rurikova, N. A. Rusakovich, H. L. Russell, J. P. Rutherfoord, N. Ruthmann, Y. F. Ryabov, M. Rybar, G. Rybkin, S. Ryu, A. Ryzhov, G. F. Rzehorz, A. F. Saavedra, G. Sabato, S. Sacerdoti, H. F-W. Sadrozinski, R. Sadykov, F. Safai Tehrani, P. Saha, M. Sahinsoy, M. Saimpert, M. Saito, T. Saito, H. Sakamoto, Y. Sakurai, G. Salamanna, J. E. Salazar Loyola, D. Salek, P. H. Sales De Bruin, D. Salihagic, A. Salnikov, J. Salt, D. Salvatore, F. Salvatore, A. Salvucci, A. Salzburger, D. Sammel, D. Sampsonidis, J. Sánchez, V. Sanchez Martinez, A. Sanchez Pineda, H. Sandaker, R. L. Sandbach, C. O. Sander, M. Sandhoff, C. Sandoval, D. P. C. Sankey, M. Sannino, A. Sansoni, C. Santoni, R. Santonico, H. Santos, I. Santoyo Castillo, K. Sapp, A. Sapronov, J. G. Saraiva, B. Sarrazin, O. Sasaki, K. Sato, E. Sauvan, G. Savage, P. Savard, N. Savic, C. Sawyer, L. Sawyer, J. Saxon, C. Sbarra, A. Sbrizzi, T. Scanlon, D. A. Scannicchio, M. Scarcella, V. Scarfone, J. Schaarschmidt, P. Schacht, B. M. Schachtner, D. Schaefer, L. Schaefer, R. Schaefer, J. Schaeffer, S. Schaepe, S. Schaetzel, U. Schäfer, A. C. Schaffer, D. Schaile, R. D. Schamberger, V. Scharf, V. A. Schegelsky, D. Scheirich, M. Schernau, C. Schiavi, S. Schier, L. K. Schildgen, C. Schillo, M. Schioppa, S. Schlenker, K. R. Schmidt-Sommerfeld, K. Schmieden, C. Schmitt, S. Schmitt, S. Schmitz, U. Schnoor, L. Schoeffel, A. Schoening, B. D. Schoenrock, E. Schopf, M. Schott, J. F. P. Schouwenberg, J. Schovancova, S. Schramm, N. Schuh, A. Schulte, M. J. Schultens, H.-C. Schultz-Coulon, H. Schulz, M. Schumacher, B. A. Schumm, Ph. Schune, A. Schwartzman, T. A. Schwarz, H. Schweiger, Ph. Schwemling, R. Schwienhorst, J. Schwindling, T. Schwindt, A. Sciandra, G. Sciolla, F. Scuri, F. Scutti, J. Searcy, P. Seema, S. C. Seidel, A. Seiden, J. M. Seixas, G. Sekhniaidze, K. Sekhon, S. J. Sekula, N. Semprini-Cesari, C. Serfon, L. Serin, L. Serkin, M. Sessa, R. Seuster, H. Severini, T. Sfiligoj, F. Sforza, A. Sfyrla, E. Shabalina, N. W. Shaikh, L. Y. Shan, R. Shang, J. T. Shank, M. Shapiro, P. B. Shatalov, K. Shaw, S. M. Shaw, A. Shcherbakova, C. Y. Shehu, Y. Shen, P. Sherwood, L. Shi, S. Shimizu, C. O. Shimmin, M. Shimojima, I. P. J. Shipsey, S. Shirabe, M. Shiyakova, J. Shlomi, A. Shmeleva, D. Shoaleh Saadi, M. J. Shochet, S. Shojaii, D. R. Shope, S. Shrestha, E. Shulga, M. A. Shupe, P. Sicho, A. M. Sickles, P. E. Sidebo, E. Sideras Haddad, O. Sidiropoulou, D. Sidorov, A. Sidoti, F. Siegert, Dj. Sijacki, J. Silva, S. B. Silverstein, V. Simak, Lj. Simic, S. Simion, E. Simioni, B. Simmons, M. Simon, P. Sinervo, N. B. Sinev, M. Sioli, G. Siragusa, I. Siral, S. Yu. Sivoklokov, J. Sjölin, M. B. Skinner, P. Skubic, M. Slater, T. Slavicek, M. Slawinska, K. Sliwa, R. Slovak, V. Smakhtin, B. H. Smart, J. Smiesko, N. Smirnov, S. Yu. Smirnov, Y. Smirnov, L. N. Smirnova, O. Smirnova, J. W. Smith, M. N. K. Smith, R. W. Smith, M. Smizanska, K. Smolek, A. A. Snesarev, I. M. Snyder, S. Snyder, R. Sobie, F. Socher, A. Soffer, D. A. Soh, G. Sokhrannyi, C. A. Solans Sanchez, M. Solar, E. Yu. Soldatov, U. Soldevila, A. A. Solodkov, A. Soloshenko, O. V. Solovyanov, V. Solovyev, P. Sommer, H. Son, H. Y. Song, A. Sopczak, D. Sosa, C. L. Sotiropoulou, R. Soualah, A. M. Soukharev, D. South, B. C. Sowden, S. Spagnolo, M. Spalla, M. Spangenberg, F. Spanò, D. Sperlich, F. Spettel, T. M. Spieker, R. Spighi, G. Spigo, L. A. Spiller, M. Spousta, R. D. St. Denis, A. Stabile, R. Stamen, S. Stamm, E. Stanecka, R. W. Stanek, C. Stanescu, M. M. Stanitzki, S. Stapnes, E. A. Starchenko, G. H. Stark, J. Stark, S. H Stark, P. Staroba, P. Starovoitov, S. Stärz, R. Staszewski, P. Steinberg, B. Stelzer, H. J. Stelzer, O. Stelzer-Chilton, H. Stenzel, G. A. Stewart, M. C. Stockton, M. Stoebe, G. Stoicea, P. Stolte, S. Stonjek, A. R. Stradling, A. Straessner, M. E. Stramaglia, J. Strandberg, S. Strandberg, A. Strandlie, M. Strauss, P. Strizenec, R. Ströhmer, D. M. Strom, R. Stroynowski, A. Strubig, S. A. Stucci, B. Stugu, N. A. Styles, D. Su, J. Su, S. Suchek, Y. Sugaya, M. Suk, V. V. Sulin, S. Sultansoy, T. Sumida, S. Sun, X. Sun, K. Suruliz, C. J. E. Suster, M. R. Sutton, S. Suzuki, M. Svatos, M. Swiatlowski, S. P. Swift, I. Sykora, T. Sykora, D. Ta, K. Tackmann, J. Taenzer, A. Taffard, R. Tafirout, N. Taiblum, H. Takai, R. Takashima, T. Takeshita, Y. Takubo, M. Talby, A. A. Talyshev, J. Tanaka, M. Tanaka, R. Tanaka, S. Tanaka, R. Tanioka, B. B. Tannenwald, S. Tapia Araya, S. Tapprogge, S. Tarem, G. F. Tartarelli, P. Tas, M. Tasevsky, T. Tashiro, E. Tassi, A. Tavares Delgado, Y. Tayalati, A. C. Taylor, G. N. Taylor, P. T. E. Taylor, W. Taylor, P. Teixeira-Dias, D. Temple, H. Ten Kate, P. K. Teng, J. J. Teoh, F. Tepel, S. Terada, K. Terashi, J. Terron, S. Terzo, M. Testa, R. J. Teuscher, T. Theveneaux-Pelzer, J. P. Thomas, J. Thomas-Wilsker, P. D. Thompson, A. S. Thompson, L. A. Thomsen, E. Thomson, M. J. Tibbetts, R. E. Ticse Torres, V. O. Tikhomirov, Yu. A. Tikhonov, S. Timoshenko, P. Tipton, S. Tisserant, K. Todome, S. Todorova-Nova, J. Tojo, S. Tokár, K. Tokushuku, E. Tolley, L. Tomlinson, M. Tomoto, L. Tompkins, K. Toms, B. Tong, P. Tornambe, E. Torrence, H. Torres, E. Torró Pastor, J. Toth, F. Touchard, D. R. Tovey, C. J. Treado, T. Trefzger, F. Tresoldi, A. Tricoli, I. M. Trigger, S. Trincaz-Duvoid, M. F. Tripiana, W. Trischuk, B. Trocmé, A. Trofymov, C. Troncon, M. Trottier-McDonald, M. Trovatelli, L. Truong, M. Trzebinski, A. Trzupek, K. W. Tsang, J. C-L. Tseng, P. V. Tsiareshka, G. Tsipolitis, N. Tsirintanis, S. Tsiskaridze, V. Tsiskaridze, E. G. Tskhadadze, K. M. Tsui, I. I. Tsukerman, V. Tsulaia, S. Tsuno, D. Tsybychev, Y. Tu, A. Tudorache, V. Tudorache, T. T. Tulbure, A. N. Tuna, S. A. Tupputi, S. Turchikhin, D. Turgeman, I. Turk Cakir, R. Turra, P. M. Tuts, G. Ucchielli, I. Ueda, M. Ughetto, F. Ukegawa, G. Unal, A. Undrus, G. Unel, F. C. Ungaro, Y. Unno, C. Unverdorben, J. Urban, P. Urquijo, P. Urrejola, G. Usai, J. Usui, L. Vacavant, V. Vacek, B. Vachon, C. Valderanis, E. Valdes Santurio, S. Valentinetti, A. Valero, L. Valéry, S. Valkar, A. Vallier, J. A. Valls Ferrer, W. Van Den Wollenberg, H. van der Graaf, N. van Eldik, P. van Gemmeren, J. Van Nieuwkoop, I. van Vulpen, M. C. van Woerden, M. Vanadia, W. Vandelli, R. Vanguri, A. Vaniachine, P. Vankov, G. Vardanyan, R. Vari, E. W. Varnes, C. Varni, T. Varol, D. Varouchas, A. Vartapetian, K. E. Varvell, J. G. Vasquez, G. A. Vasquez, F. Vazeille, T. Vazquez Schroeder, J. Veatch, V. Veeraraghavan, L. M. Veloce, F. Veloso, S. Veneziano, A. Ventura, M. Venturi, N. Venturi, A. Venturini, V. Vercesi, M. Verducci, W. Verkerke, J. C. Vermeulen, M. C. Vetterli, N. Viaux Maira, O. Viazlo, I. Vichou, T. Vickey, O. E. Vickey Boeriu, G. H. A. Viehhauser, S. Viel, L. Vigani, M. Villa, M. Villaplana Perez, E. Vilucchi, M. G. Vincter, V. B. Vinogradov, A. Vishwakarma, C. Vittori, I. Vivarelli, S. Vlachos, M. Vlasak, M. Vogel, P. Vokac, G. Volpi, M. Volpi, H. von der Schmitt, E. von Toerne, V. Vorobel, K. Vorobev, M. Vos, R. Voss, J. H. Vossebeld, N. Vranjes, M. Vranjes Milosavljevic, V. Vrba, M. Vreeswijk, R. Vuillermet, I. Vukotic, P. Wagner, W. Wagner, J. Wagner-Kuhr, H. Wahlberg, S. Wahrmund, J. Wakabayashi, J. Walder, R. Walker, W. Walkowiak, V. Wallangen, C. Wang, C. Wang, F. Wang, H. Wang, H. Wang, J. Wang, J. Wang, Q. Wang, R. Wang, S. M. Wang, T. Wang, W. Wang, W. Wang, Z. Wang, C. Wanotayaroj, A. Warburton, C. P. Ward, D. R. Wardrope, A. Washbrook, P. M. Watkins, A. T. Watson, M. F. Watson, G. Watts, S. Watts, B. M. Waugh, A. F. Webb, S. Webb, M. S. Weber, S. W. Weber, S. A. Weber, J. S. Webster, A. R. Weidberg, B. Weinert, J. Weingarten, C. Weiser, H. Weits, P. S. Wells, T. Wenaus, T. Wengler, S. Wenig, N. Wermes, M. D. Werner, P. Werner, M. Wessels, K. Whalen, N. L. Whallon, A. M. Wharton, A. White, M. J. White, R. White, D. Whiteson, F. J. Wickens, W. Wiedenmann, M. Wielers, C. Wiglesworth, L. A. M. Wiik-Fuchs, A. Wildauer, F. Wilk, H. G. Wilkens, H. H. Williams, S. Williams, C. Willis, S. Willocq, J. A. Wilson, I. Wingerter-Seez, E. Winkels, F. Winklmeier, O. J. Winston, B. T. Winter, M. Wittgen, M. Wobisch, T. M. H. Wolf, R. Wolff, M. W. Wolter, H. Wolters, S. D. Worm, B. K. Wosiek, J. Wotschack, M. J. Woudstra, K. W. Wozniak, M. Wu, S. L. Wu, X. Wu, Y. Wu, T. R. Wyatt, B. M. Wynne, S. Xella, Z. Xi, L. Xia, D. Xu, L. Xu, B. Yabsley, S. Yacoob, D. Yamaguchi, Y. Yamaguchi, A. Yamamoto, S. Yamamoto, T. Yamanaka, K. Yamauchi, Y. Yamazaki, Z. Yan, H. Yang, H. Yang, Y. Yang, Z. Yang, W-M. Yao, Y. C. Yap, Y. Yasu, E. Yatsenko, K. H. Yau Wong, J. Ye, S. Ye, I. Yeletskikh, E. Yigitbasi, E. Yildirim, K. Yorita, K. Yoshihara, C. Young, C. J. S. Young, D. R. Yu, J. Yu, J. Yu, S. P. Y. Yuen, I. Yusuff, B. Zabinski, G. Zacharis, R. Zaidan, A. M. Zaitsev, N. Zakharchuk, J. Zalieckas, A. Zaman, S. Zambito, D. Zanzi, C. Zeitnitz, M. Zeman, A. Zemla, J. C. Zeng, Q. Zeng, O. Zenin, T. Ženiš, D. Zerwas, D. Zhang, F. Zhang, G. Zhang, H. Zhang, J. Zhang, L. Zhang, L. Zhang, M. Zhang, R. Zhang, R. Zhang, X. Zhang, Y. Zhang, Z. Zhang, X. Zhao, Y. Zhao, Z. Zhao, A. Zhemchugov, J. Zhong, B. Zhou, C. Zhou, L. Zhou, M. Zhou, M. Zhou, N. Zhou, C. G. Zhu, H. Zhu, J. Zhu, Y. Zhu, X. Zhuang, K. Zhukov, A. Zibell, D. Zieminska, N. I. Zimine, C. Zimmermann, S. Zimmermann, Z. Zinonos, M. Zinser, M. Ziolkowski, L. Živković, G. Zobernig, A. Zoccoli, R. Zou, M. zur Nedden, L. Zwalinski, ATLAS Collaboration

**Affiliations:** 1 Department of Physics, University of Adelaide, Adelaide, Australia; 2 Physics Department, SUNY Albany, Albany, NY USA; 3 Department of Physics, University of Alberta, Edmonton, AB Canada; 4 Department of Physics, Ankara University, Ankara, Turkey; 5 Istanbul Aydin University, Istanbul, Turkey; 6 Division of Physics, TOBB University of Economics and Technology, Ankara, Turkey; 7 LAPP, CNRS/IN2P3 and Université Savoie Mont Blanc, Annecy-le-Vieux, France; 8 High Energy Physics Division, Argonne National Laboratory, Argonne, IL USA; 9 Department of Physics, University of Arizona, Tucson, AZ USA; 10 Department of Physics, The University of Texas at Arlington, Arlington, TX USA; 11 Physics Department, National and Kapodistrian University of Athens, Athens, Greece; 12 Physics Department, National Technical University of Athens, Zografou, Greece; 13 Department of Physics, The University of Texas at Austin, Austin, TX USA; 14 Institute of Physics, Azerbaijan Academy of Sciences, Baku, Azerbaijan; 15 Institut de Física d’Altes Energies (IFAE), The Barcelona Institute of Science and Technology, Barcelona, Spain; 16 Institute of Physics, University of Belgrade, Belgrade, Serbia; 17 Department for Physics and Technology, University of Bergen, Bergen, Norway; 18 Physics Division, Lawrence Berkeley National Laboratory, University of California, Berkeley, CA USA; 19 Department of Physics, Humboldt University, Berlin, Germany; 20 Albert Einstein Center for Fundamental Physics, Laboratory for High Energy Physics, University of Bern, Bern, Switzerland; 21 School of Physics and Astronomy, University of Birmingham, Birmingham, UK; 22 Department of Physics, Bogazici University, Istanbul, Turkey; 23 Department of Physics Engineering, Gaziantep University, Gaziantep, Turkey; 24 Faculty of Engineering and Natural Sciences, Istanbul Bilgi University, Istanbul, Turkey; 25 Faculty of Engineering and Natural Sciences, Bahcesehir University, Istanbul, Turkey; 26 Centro de Investigaciones, Universidad Antonio Narino, Bogotá, Colombia; 27 INFN Sezione di Bologna, Bologna, Italy; 28 Dipartimento di Fisica e Astronomia, Università di Bologna, Bologna, Italy; 29 Physikalisches Institut, University of Bonn, Bonn, Germany; 30 Department of Physics, Boston University, Boston, MA USA; 31 Department of Physics, Brandeis University, Waltham, MA USA; 32 Universidade Federal do Rio De Janeiro COPPE/EE/IF, Rio de Janeiro, Brazil; 33 Electrical Circuits Department, Federal University of Juiz de Fora (UFJF), Juiz de Fora, Brazil; 34 Federal University of Sao Joao del Rei (UFSJ), Sao Joao del Rei, Brazil; 35 Instituto de Fisica, Universidade de Sao Paulo, São Paulo, Brazil; 36 Physics Department, Brookhaven National Laboratory, Upton, NY USA; 37 Transilvania University of Brasov, Brasov, Romania; 38 Horia Hulubei National Institute of Physics and Nuclear Engineering, Bucharest, Romania; 39 Department of Physics, Alexandru Ioan Cuza University of Iasi, Iasi, Romania; 40 Physics Department, National Institute for Research and Development of Isotopic and Molecular Technologies, Cluj-Napoca, Romania; 41 University Politehnica Bucharest, Bucharest, Romania; 42 West University in Timisoara, Timisoara, Romania; 43 Departamento de Física, Universidad de Buenos Aires, Buenos Aires, Argentina; 44 Cavendish Laboratory, University of Cambridge, Cambridge, UK; 45 Department of Physics, Carleton University, Ottawa, ON Canada; 46 CERN, Geneva, Switzerland; 47 Enrico Fermi Institute, University of Chicago, Chicago, IL USA; 48 Departamento de Física, Pontificia Universidad Católica de Chile, Santiago, Chile; 49 Departamento de Física, Universidad Técnica Federico Santa María, Valparaiso, Chile; 50 Institute of High Energy Physics, Chinese Academy of Sciences, Beijing, China; 51 Department of Physics, Nanjing University, Jiangsu, China; 52 Physics Department, Tsinghua University, Beijing, 100084 China; 53 Department of Modern Physics, University of Science and Technology of China, Anhui, China; 54 School of Physics, Shandong University, Shandong, China; 55 Department of Physics and Astronomy, Key Laboratory for Particle Physics, Astrophysics and Cosmology, Ministry of Education, Shanghai Key Laboratory for Particle Physics and Cosmology, Shanghai Jiao Tong University, Shanghai (also PKU-CHEP), Shanghai, China; 56 Université Clermont Auvergne, CNRS/IN2P3, LPC, Clermont-Ferrand, France; 57 Nevis Laboratory, Columbia University, Irvington, NY USA; 58 Niels Bohr Institute, University of Copenhagen, Kopenhavn, Denmark; 59 INFN Gruppo Collegato di Cosenza, Laboratori Nazionali di Frascati, Frascati, Italy; 60 Dipartimento di Fisica, Università della Calabria, Rende, Italy; 61 Faculty of Physics and Applied Computer Science, AGH University of Science and Technology, Kraków, Poland; 62 Marian Smoluchowski Institute of Physics, Jagiellonian University, Kraków, Poland; 63 Institute of Nuclear Physics, Polish Academy of Sciences, Kraków, Poland; 64 Physics Department, Southern Methodist University, Dallas, TX USA; 65 Physics Department, University of Texas at Dallas, Richardson, TX USA; 66 DESY, Hamburg and Zeuthen, Germany; 67 Lehrstuhl für Experimentelle Physik IV, Technische Universität Dortmund, Dortmund, Germany; 68 Institut für Kern- und Teilchenphysik, Technische Universität Dresden, Dresden, Germany; 69 Department of Physics, Duke University, Durham, NC USA; 70 SUPA-School of Physics and Astronomy, University of Edinburgh, Edinburgh, UK; 71 INFN Laboratori Nazionali di Frascati, Frascati, Italy; 72 Fakultät für Mathematik und Physik, Albert-Ludwigs-Universität, Freiburg, Germany; 73 Departement de Physique Nucleaire et Corpusculaire, Université de Genève, Geneva, Switzerland; 74 INFN Sezione di Genova, Genoa, Italy; 75 Dipartimento di Fisica, Università di Genova, Genoa, Italy; 76 E. Andronikashvili Institute of Physics, Iv. Javakhishvili Tbilisi State University, Tbilisi, Georgia; 77 High Energy Physics Institute, Tbilisi State University, Tbilisi, Georgia; 78 II Physikalisches Institut, Justus-Liebig-Universität Giessen, Giessen, Germany; 79 SUPA-School of Physics and Astronomy, University of Glasgow, Glasgow, UK; 80 II Physikalisches Institut, Georg-August-Universität, Göttingen, Germany; 81 Laboratoire de Physique Subatomique et de Cosmologie, Université Grenoble-Alpes, CNRS/IN2P3, Grenoble, France; 82 Laboratory for Particle Physics and Cosmology, Harvard University, Cambridge, MA USA; 83 Kirchhoff-Institut für Physik, Ruprecht-Karls-Universität Heidelberg, Heidelberg, Germany; 84 Physikalisches Institut, Ruprecht-Karls-Universität Heidelberg, Heidelberg, Germany; 85 ZITI Institut für technische Informatik, Ruprecht-Karls-Universität Heidelberg, Mannheim, Germany; 86 Faculty of Applied Information Science, Hiroshima Institute of Technology, Hiroshima, Japan; 87 Department of Physics, The Chinese University of Hong Kong, Shatin, NT Hong Kong; 88 Department of Physics, The University of Hong Kong, Hong Kong, China; 89 Department of Physics, Institute for Advanced Study, The Hong Kong University of Science and Technology, Clear Water Bay, Kowloon, Hong Kong, China; 90 Department of Physics, National Tsing Hua University, Taiwan, Taiwan; 91 Department of Physics, Indiana University, Bloomington, IN USA; 92 Institut für Astro- und Teilchenphysik, Leopold-Franzens-Universität, Innsbruck, Austria; 93 University of Iowa, Iowa City, IA USA; 94 Department of Physics and Astronomy, Iowa State University, Ames, IA USA; 95 Joint Institute for Nuclear Research, JINR Dubna, Dubna, Russia; 96 KEK, High Energy Accelerator Research Organization, Tsukuba, Japan; 97 Graduate School of Science, Kobe University, Kobe, Japan; 98 Faculty of Science, Kyoto University, Kyoto, Japan; 99 Kyoto University of Education, Kyoto, Japan; 100 Department of Physics, Kyushu University, Fukuoka, Japan; 101 Instituto de Física La Plata, Universidad Nacional de La Plata and CONICET, La Plata, Argentina; 102 Physics Department, Lancaster University, Lancaster, UK; 103 INFN Sezione di Lecce, Lecce, Italy; 104 Dipartimento di Matematica e Fisica, Università del Salento, Lecce, Italy; 105 Oliver Lodge Laboratory, University of Liverpool, Liverpool, UK; 106 Department of Experimental Particle Physics, Jožef Stefan Institute and Department of Physics, University of Ljubljana, Ljubljana, Slovenia; 107 School of Physics and Astronomy, Queen Mary University of London, London, UK; 108 Department of Physics, Royal Holloway University of London, Surrey, UK; 109 Department of Physics and Astronomy, University College London, London, UK; 110 Louisiana Tech University, Ruston, LA USA; 111 Laboratoire de Physique Nucléaire et de Hautes Energies, UPMC and Université Paris-Diderot and CNRS/IN2P3, Paris, France; 112 Fysiska institutionen, Lunds universitet, Lund, Sweden; 113 Departamento de Fisica Teorica C-15, Universidad Autonoma de Madrid, Madrid, Spain; 114 Institut für Physik, Universität Mainz, Mainz, Germany; 115 School of Physics and Astronomy, University of Manchester, Manchester, UK; 116 CPPM, Aix-Marseille Université and CNRS/IN2P3, Marseille, France; 117 Department of Physics, University of Massachusetts, Amherst, MA USA; 118 Department of Physics, McGill University, Montreal, QC Canada; 119 School of Physics, University of Melbourne, Victoria, Australia; 120 Department of Physics, The University of Michigan, Ann Arbor, MI USA; 121 Department of Physics and Astronomy, Michigan State University, East Lansing, MI USA; 122 INFN Sezione di Milano, Milan, Italy; 123 Dipartimento di Fisica, Università di Milano, Milan, Italy; 124 B.I. Stepanov Institute of Physics, National Academy of Sciences of Belarus, Minsk, Republic of Belarus; 125 Research Institute for Nuclear Problems of Byelorussian State University, Minsk, Republic of Belarus; 126 Group of Particle Physics, University of Montreal, Montreal, QC Canada; 127 P.N. Lebedev Physical Institute of the Russian Academy of Sciences, Moscow, Russia; 128 Institute for Theoretical and Experimental Physics (ITEP), Moscow, Russia; 129 National Research Nuclear University MEPhI, Moscow, Russia; 130 D.V. Skobeltsyn Institute of Nuclear Physics, M.V. Lomonosov Moscow State University, Moscow, Russia; 131 Fakultät für Physik, Ludwig-Maximilians-Universität München, Munich, Germany; 132 Max-Planck-Institut für Physik (Werner-Heisenberg-Institut), Munich, Germany; 133 Nagasaki Institute of Applied Science, Nagasaki, Japan; 134 Graduate School of Science and Kobayashi-Maskawa Institute, Nagoya University, Nagoya, Japan; 135 INFN Sezione di Napoli, Naples, Italy; 136 Dipartimento di Fisica, Università di Napoli, Naples, Italy; 137 Department of Physics and Astronomy, University of New Mexico, Albuquerque, NM USA; 138 Institute for Mathematics, Astrophysics and Particle Physics, Radboud University Nijmegen/Nikhef, Nijmegen, The Netherlands; 139 Nikhef National Institute for Subatomic Physics, University of Amsterdam, Amsterdam, The Netherlands; 140 Department of Physics, Northern Illinois University, DeKalb, IL USA; 141 Budker Institute of Nuclear Physics, SB RAS, Novosibirsk, Russia; 142 Department of Physics, New York University, New York, NY USA; 143 Ohio State University, Columbus, OH USA; 144 Faculty of Science, Okayama University, Okayama, Japan; 145 Homer L. Dodge Department of Physics and Astronomy, University of Oklahoma, Norman, OK USA; 146 Department of Physics, Oklahoma State University, Stillwater, OK USA; 147 Palacký University, RCPTM, Olomouc, Czech Republic; 148 Center for High Energy Physics, University of Oregon, Eugene, OR USA; 149 LAL, Univ. Paris-Sud, CNRS/IN2P3, Université Paris-Saclay, Orsay, France; 150 Graduate School of Science, Osaka University, Osaka, Japan; 151 Department of Physics, University of Oslo, Oslo, Norway; 152 Department of Physics, Oxford University, Oxford, UK; 153 INFN Sezione di Pavia, Pavia, Italy; 154 Dipartimento di Fisica, Università di Pavia, Pavia, Italy; 155 Department of Physics, University of Pennsylvania, Philadelphia, PA USA; 156 National Research Centre “Kurchatov Institute” B.P. Konstantinov Petersburg Nuclear Physics Institute, St. Petersburg, Russia; 157 INFN Sezione di Pisa, Pisa, Italy; 158 Dipartimento di Fisica E. Fermi, Università di Pisa, Pisa, Italy; 159 Department of Physics and Astronomy, University of Pittsburgh, Pittsburgh, PA USA; 160 Laboratório de Instrumentação e Física Experimental de Partículas-LIP, Lisbon, Portugal; 161 Faculdade de Ciências, Universidade de Lisboa, Lisbon, Portugal; 162 Department of Physics, University of Coimbra, Coimbra, Portugal; 163 Centro de Física Nuclear da Universidade de Lisboa, Lisbon, Portugal; 164 Departamento de Fisica, Universidade do Minho, Braga, Portugal; 165 Departamento de Fisica Teorica y del Cosmos and CAFPE, Universidad de Granada, Granada, Spain; 166 Dep Fisica and CEFITEC of Faculdade de Ciencias e Tecnologia, Universidade Nova de Lisboa, Caparica, Portugal; 167 Institute of Physics, Academy of Sciences of the Czech Republic, Praha, Czech Republic; 168 Czech Technical University in Prague, Praha, Czech Republic; 169 Faculty of Mathematics and Physics, Charles University, Prague, Czech Republic; 170 State Research Center Institute for High Energy Physics (Protvino), NRC KI, Protvino, Russia; 171 Particle Physics Department, Rutherford Appleton Laboratory, Didcot, UK; 172 INFN Sezione di Roma, Rome, Italy; 173 Dipartimento di Fisica, Sapienza Università di Roma, Rome, Italy; 174 INFN Sezione di Roma Tor Vergata, Rome, Italy; 175 Dipartimento di Fisica, Università di Roma Tor Vergata, Rome, Italy; 176 INFN Sezione di Roma Tre, Rome, Italy; 177 Dipartimento di Matematica e Fisica, Università Roma Tre, Rome, Italy; 178 Faculté des Sciences Ain Chock, Réseau Universitaire de Physique des Hautes Energies-Université Hassan II, Casablanca, Morocco; 179 Centre National de l’Energie des Sciences Techniques Nucleaires, Rabat, Morocco; 180 Faculté des Sciences Semlalia, Université Cadi Ayyad, LPHEA-Marrakech, Marrakech, Morocco; 181 Faculté des Sciences, Université Mohamed Premier and LPTPM, Oujda, Morocco; 182 Faculté des Sciences, Université Mohammed V, Rabat, Morocco; 183 DSM/IRFU (Institut de Recherches sur les Lois Fondamentales de l’Univers), CEA Saclay (Commissariat à l’Energie Atomique et aux Energies Alternatives), Gif-sur-Yvette, France; 184 Santa Cruz Institute for Particle Physics, University of California Santa Cruz, Santa Cruz, CA USA; 185 Department of Physics, University of Washington, Seattle, WA USA; 186 Department of Physics and Astronomy, University of Sheffield, Sheffield, UK; 187 Department of Physics, Shinshu University, Nagano, Japan; 188 Department Physik, Universität Siegen, Siegen, Germany; 189 Department of Physics, Simon Fraser University, Burnaby, BC Canada; 190 SLAC National Accelerator Laboratory, Stanford, CA USA; 191 Faculty of Mathematics, Physics and Informatics, Comenius University, Bratislava, Slovak Republic; 192 Department of Subnuclear Physics, Institute of Experimental Physics of the Slovak Academy of Sciences, Kosice, Slovak Republic; 193 Department of Physics, University of Cape Town, Cape Town, South Africa; 194 Department of Physics, University of Johannesburg, Johannesburg, South Africa; 195 School of Physics, University of the Witwatersrand, Johannesburg, South Africa; 196 Department of Physics, Stockholm University, Stockholm, Sweden; 197 The Oskar Klein Centre, Stockholm, Sweden; 198 Physics Department, Royal Institute of Technology, Stockholm, Sweden; 199 Departments of Physics and Astronomy and Chemistry, Stony Brook University, Stony Brook, NY USA; 200 Department of Physics and Astronomy, University of Sussex, Brighton, UK; 201 School of Physics, University of Sydney, Sydney, Australia; 202 Institute of Physics, Academia Sinica, Taipei, Taiwan; 203 Department of Physics, Technion: Israel Institute of Technology, Haifa, Israel; 204 Raymond and Beverly Sackler School of Physics and Astronomy, Tel Aviv University, Tel Aviv, Israel; 205 Department of Physics, Aristotle University of Thessaloniki, Thessaloniki, Greece; 206 International Center for Elementary Particle Physics and Department of Physics, The University of Tokyo, Tokyo, Japan; 207 Graduate School of Science and Technology, Tokyo Metropolitan University, Tokyo, Japan; 208 Department of Physics, Tokyo Institute of Technology, Tokyo, Japan; 209 Tomsk State University, Tomsk, Russia; 210 Department of Physics, University of Toronto, Toronto, ON Canada; 211 INFN-TIFPA, Trento, Italy; 212 University of Trento, Trento, Italy; 213 TRIUMF, Vancouver, BC Canada; 214 Department of Physics and Astronomy, York University, Toronto, ON Canada; 215 Faculty of Pure and Applied Sciences, and Center for Integrated Research in Fundamental Science and Engineering, University of Tsukuba, Tsukuba, Japan; 216 Department of Physics and Astronomy, Tufts University, Medford, MA USA; 217 Department of Physics and Astronomy, University of California Irvine, Irvine, CA USA; 218 INFN Gruppo Collegato di Udine, Sezione di Trieste, Udine, Italy; 219 ICTP, Trieste, Italy; 220 Dipartimento di Chimica, Fisica e Ambiente, Università di Udine, Udine, Italy; 221 Department of Physics and Astronomy, University of Uppsala, Uppsala, Sweden; 222 Department of Physics, University of Illinois, Urbana, IL USA; 223 Instituto de Fisica Corpuscular (IFIC) and Departamento de Fisica Atomica, Molecular y Nuclear and Departamento de Ingeniería Electrónica and Instituto de Microelectrónica de Barcelona (IMB-CNM), University of Valencia and CSIC, Valencia, Spain; 224 Department of Physics, University of British Columbia, Vancouver, BC Canada; 225 Department of Physics and Astronomy, University of Victoria, Victoria, BC Canada; 226 Department of Physics, University of Warwick, Coventry, UK; 227 Waseda University, Tokyo, Japan; 228 Department of Particle Physics, The Weizmann Institute of Science, Rehovot, Israel; 229 Department of Physics, University of Wisconsin, Madison, WI USA; 230 Fakultät für Physik und Astronomie, Julius-Maximilians-Universität, Würzburg, Germany; 231 Fakultät für Mathematik und Naturwissenschaften, Fachgruppe Physik, Bergische Universität Wuppertal, Wuppertal, Germany; 232 Department of Physics, Yale University, New Haven, CT USA; 233 Yerevan Physics Institute, Yerevan, Armenia; 234 CH-1211, Geneva 23, Switzerland; 235 Centre de Calcul de l’Institut National de Physique Nucléaire et de Physique des Particules (IN2P3), Villeurbanne, France; 236 CERN, 1211 Geneva 23, Switzerland

## Abstract

The distributions of transverse momentum and longitudinal momentum fraction of charged particles in jets are measured in Pb+Pb and *pp* collisions with the ATLAS detector at the LHC. The distributions are measured as a function of jet transverse momentum and rapidity. The analysis utilises an integrated luminosity of 0.14 nb$^{-1}$ of Pb+Pb data and 4.0 pb$^{-1}$ of *pp* data collected in 2011 and 2013, respectively, at the same centre-of-mass energy of 2.76 TeV per colliding nucleon pair. The distributions measured in *pp* collisions are used as a reference for those measured in Pb+Pb collisions in order to evaluate the impact on the internal structure of jets from the jet energy loss of fast partons propagating through the hot, dense medium created in heavy-ion collisions. Modest but significant centrality-dependent modifications of fragmentation functions in Pb+Pb collisions with respect to those in *pp* collisions are seen. No significant dependence of modifications on jet $p_\mathrm{T}$ and rapidity selections is observed except for the fragments with the highest transverse momenta for which some reduction of yields is observed for more forward jets.

## Introduction

Heavy-ion collisions at ultra-relativistic energies produce a medium of strongly interacting nuclear matter composed of deconfined colour charges which is commonly called a quark–gluon plasma (QGP) [1–4]. Hard-scattering processes occurring in these collisions produce high transverse momentum, $p_{\mathrm{T}}$, partons that propagate through the medium and lose energy. This phenomenon is termed “jet quenching”. More specifically, jet quenching is a process in which constituents of the parton shower may be elastically or inelastically scattered by the constituents of the plasma, resulting in the suppression of jet production and the modification of the internal structure of jets [5–7]. Inclusive-jet suppression has been measured previously at the LHC in terms of the nuclear modification factor [8–12]. A suppression of jet production by about a factor of two in central heavy-ion collisions was observed. The internal structure of jets was also measured [13–16] and these measurements revealed modification of the distributions of the jet fragments. The measurements of the jet structure were supplemented by a measurement of the correlation of the jet suppression with missing transverse momentum [17], leading to a conclusion that the energy lost by partons is transferred predominantly to soft particles being radiated at large angles with respect to the direction of the original parton.

This paper presents a new measurement of the internal structure of jets by ATLAS in Pb+Pb and *pp* collisions, both at the same centre-of-mass energy per colliding nucleon pair of 2.76 $\text {TeV}$. The measurement utilised Pb+Pb data collected during 2011 corresponding to an integrated luminosity of 0.14 nb$^{-1}$ as well as data from *pp* collisions recorded during 2013 corresponding to 4.0 pb$^{-1}$. In this paper the same quantities that were introduced in Ref. [13] are used, namely the jet fragmentation functions, *D*(*z*), and distributions of charged-particle transverse momenta measured inside the jet, $D(p_{\mathrm{T}})$. The *D*(*z*) distributions are defined as

1 $$\begin{aligned} D(z)\equiv \dfrac{1}{N_{\mathrm{jet}}} \dfrac{\mathrm {d}N_{\mathrm{ch}}}{\mathrm {d}z}, \end{aligned}$$

where $N_{\mathrm{jet}}$ is the total number of jets, $N_{\mathrm{ch}}$ is the number of charged particles associated with a jet, and the longitudinal momentum fraction *z* is defined as

2 $$\begin{aligned} z \equiv \frac{p_{\mathrm{T}}}{p_{\mathrm{T}}^{\mathrm{jet}}} \cos \Delta R = \frac{p_{\mathrm{T}}}{p_{\mathrm{T}}^{\mathrm{jet}}} \cos {\sqrt{(\Delta \eta )^2 + (\Delta \phi )^2}}. \end{aligned}$$

Here $p_{\mathrm{T}}^{\mathrm{jet}}$ is the transverse momentum of a jet measured with respect to the beam direction, $p_{\mathrm{T}}$ stands for the transverse momentum of a charged particle, $\Delta \eta$ and $\Delta \phi$ are the distance between the jet axis and the charged-particle direction in pseudorapidity and azimuth,^1^ respectively.^2^ The $D(p_{\mathrm{T}})$ distributions are defined as

3 $$\begin{aligned} D(p_{\mathrm{T}}) \equiv \frac{1}{N_{\mathrm{jet}}} \frac{\mathrm{d}N_{\mathrm{ch}}(p_{\text {T}})}{\mathrm{d}p_{\text {T}}} . \end{aligned}$$

The fragmentation distributions were measured for jets reconstructed with the anti-$k_{t}$ algorithm [18] with the radius parameter set to $R=0.4$. The charged particles were matched to a jet by requiring the distance between the jet axis and the charged particle to be $\Delta R < 0.4$. The fragmentation distributions were fully corrected to the particle level.

In the first measurement of jet fragmentation by ATLAS in heavy-ion collisions [13], the measurements were performed for jets with the radius parameters $R=0.2,0.3,$ and 0.4. Jet fragments having a minimum $p_{\mathrm{T}}$ of $2~\text {GeV}$ were measured within an angular range $\Delta R = 0.4$ from the jet axis. The *D*(*z*) and $D(p_{\mathrm{T}})$ distributions were presented for seven bins in collision centrality. Ratios of fragmentation functions in the different centrality bins to the 60–80% bin were presented and used to evaluate the modifications of the jet fragmentation caused by the medium. Those ratios exhibited an enhancement in fragment yield in central collisions for $z \lesssim 0.04$, a reduction in fragment yield for $0.04 \lesssim z \lesssim 0.2$, and an enhancement in the fragment yield for $z > 0.4$. The modifications were found to decrease monotonically with decreasing collision centrality from 0–10 to 50–60%. A similar set of modifications was observed in the $D(p_{\mathrm{T}})$ distributions over corresponding $p_{\text {T}}$ ranges.

This new analysis provides a measurement of the jet structure of $R = 0.4$ jets using the same observables, but it decreases the minimum $p_{\mathrm{T}}$ for charged particles to 1 $\text {GeV}$ and evaluates the fragmentation observables differentially in jet $p_{\mathrm{T}}$ and *y*. Furthermore, the new analysis uses the fragment distributions measured in *pp* collisions as a reference for the measurement of jet fragmentation in heavy-ion collisions. Using this information about the jet structure, the flow of the quenched jet energy and number of charged particles was quantified as a function of the centrality.

The content of this paper is organised as follows: Sect. 2 describes the experimental set-up. Section 3 describes the event selection and data sets. The jet and track reconstruction and selection are introduced in Sect. 4. Section 5 discusses the analysis procedure. The estimation of systematic uncertainties is given is Sect. 6. Section 7 describes the results of the measurement. Section 8 provides a discussion of the results, and Sect. 9 summarises the analysis.

## Experimental set-up

The measurements presented in this paper were performed using the ATLAS calorimeter, inner detector, trigger, and data acquisition systems [19]. The ATLAS calorimeter system consists of a liquid argon (LAr) electromagnetic (EM) calorimeter covering $|\eta |<3.2$, a steel–scintillator sampling hadronic calorimeter covering $|\eta | < 1.7$, a LAr hadronic calorimeter covering $1.5< |\eta | < 3.2$, and a LAr forward calorimeter (FCal) covering $3.2< |\eta | < 4.9$. The hadronic calorimeter has three sampling layers, longitudinal in shower depth, and has a $\Delta \eta \times \Delta \phi$ granularity of $0.1 \times \pi /32$ for $|\eta | < 2.5$ and $0.2 \times 2\pi /32$ for $2.5< |\eta | < 4.9$.^3^ The EM calorimeters are longitudinally segmented in shower depth into three compartments with an additional pre-sampler layer. The EM calorimeter has a granularity that varies with layer and pseudorapidity, but which is generally much finer than that of the hadronic calorimeter. The middle sampling layer, which typically has the largest energy deposit in EM showers, has a granularity of $0.025 \times 0.0245$ over $|\eta | < 2.5$.

The inner detector [20] measures charged particles within the pseudorapidity interval $|\eta |<2.5$ using a combination of silicon pixel detectors, silicon microstrip detectors (SCT), and a straw-tube transition radiation tracker (TRT), all immersed in a 2 T axial magnetic field. All three detectors are composed of a barrel and two symmetrically placed end-cap sections. The pixel detector is composed of three layers of sensors with nominal feature size $50 \times 400~{\upmu \mathrm{m}}$. The microstrip detector’s barrel section contains four layers of modules with 80 $\upmu \mathrm {m}$ pitch sensors on both sides, while the end-caps consist of nine layers of double-sided modules with radial strips having a mean pitch of $80~\upmu \mathrm {m}$. The two sides of each layer in both the barrel and the end-caps have a relative stereo angle of 40 mrad. The transition radiation tracker contains up to 73 (160) layers of staggered straws interleaved with fibres in the barrel (end-cap). Charged particles with $p_{\text {T}} \gtrsim 0.5$ $\text {GeV}$ and $|\eta | < 2.5$ typically traverse three layers of silicon pixel detectors, four layers of double-sided microstrip sensors, and 36 straws if $|\eta | < 2.0$.

Minimum-bias Pb+Pb collisions were selected using measurements from the zero-degree calorimeters (ZDCs) and the minimum-bias trigger scintillator (MBTS) counters [19]. The ZDCs are located symmetrically at a longitudinal distance of $\pm 140$ m from the detector centre and cover $|\eta | > 8.3$. In Pb+Pb collisions, the ZDCs primarily measure “spectator” neutrons, which originate from the incident nuclei and do not interact hadronically. The MBTS detects charged particles over $2.1< |\eta | < 3.9$ using two counters placed at a distance of $\pm 3.6$ m from the interaction point. Each counter is divided into 16 modules with 8 different positions in $\eta$ and $\phi$. Each counter provides measurement of both the pulse heights and arrival times of ionisation energy deposits.

## Event selection and data sets

The analysis utilised an integrated luminosity of 0.14 nb$^{-1}$ of Pb+Pb data and 4.0 pb$^{-1}$ of *pp* data collected in 2011 and 2013, respectively. The Pb+Pb events used in the analysis were required to have a reconstructed primary vertex and a time difference between hits from the two sides of the MBTS detector of less than 3 ns. The primary vertices were reconstructed from charged-particle tracks with $p_{\text {T}} > 0.5~\text {GeV}$. The tracks were reconstructed from hits in the inner detector using the standard track-reconstruction algorithm [21] with settings optimised for the high hit density in heavy-ion collisions [22]. The Pb+Pb events were selected for recording by a combination of Level-1 minimum-bias and high level trigger (HLT) jet triggers. The Level-1 trigger required a total transverse energy measured in the calorimeter of greater than 10 $\text {GeV}$. The HLT jet trigger ran the offline Pb+Pb jet-reconstruction algorithm, described below, for $R= 0.2$ jets except for the application of the final hadronic energy-scale correction. The HLT selected events containing an $R= 0.2$ jet with transverse energy $E_{\text {T}} > 20$ $\text {GeV}$ in the $|\eta | < 3.2$ range. A total of 14.2 million events satisfied these event selection criteria. The performance of the jet triggering is summarised in Ref. [23].

The centrality of Pb+Pb collisions was characterised by $\Sigma E_{\mathrm{T}}^{\mathrm{FCal}}$, the total transverse energy measured in the FCal [22]. The results in this paper were obtained using seven centrality bins defined according to successive percentiles of the $\Sigma E_{\mathrm{T}}^{\mathrm{FCal}}$ distribution ordered from the most central, highest $\Sigma E_{\mathrm{T}}^{\mathrm{FCal}}$, to the most peripheral collisions: 0–10, 10–20, 20–30, 30–40, 40–50, 50–60, and 60–80%. The percentiles were defined after correcting the $\Sigma E_{\mathrm{T}}^{\mathrm{FCal}}$ distribution for the 2% minimum-bias trigger inefficiency which only affects the most peripheral collisions (80–100%), that were not included in this analysis.

The *pp* events used in the analysis were selected using the ATLAS jet trigger [24] with a requirement of a minimum jet $p_{\mathrm{T}}$ of 75 $\text {GeV}$. The *pp* events were required to contain at least one primary vertex, reconstructed from at least two tracks with $p_{\mathrm{T}}>0.5~\text {GeV}$. Jets originating from all selected events were included in the measurement.

The performance of the ATLAS detector and offline analysis in measuring jets and charged particles in *pp* collisions was evaluated using a sample of 15 million Monte Carlo (MC) events obtained from PYTHIA [25] hard-scattering events (using PYTHIA version 6.425, with parameter values set to the AUET2B tune [26], and CTEQ6L1 parton distribution functions [27]). The generator-level spectrum of $R=0.4$ jets covers the transverse momentum interval of $20< p_{\mathrm{T}}< 500~\text {GeV}$, which is sufficient to cover the jet $p_{\mathrm{T}}$ range in the data. The detector effects were fully simulated [28] using GEANT4 [29]. The reconstruction performance in Pb+Pb collisions was evaluated using a sample of 18 million events obtained by overlaying simulated PYTHIA hard-scattering events onto minimum-bias Pb+Pb events recorded in 2011. In this overlay procedure, the simulated hits were combined with the data from minimum-bias events to produce the final sample. The generator-level spectrum of jets in the overlay sample covers the transverse momentum interval of $35< p_{\mathrm{T}}< 560~\text {GeV}$. In all samples, the generator-level charged particles are defined as all final-state charged PYTHIA particles with lifetimes longer than $0.3 \times 10^{-10}$ s originating from the primary interaction or from the subsequent decay of particles with shorter lifetimes.^4^

## Jet and track selection

Jets were reconstructed using the techniques described in Ref. [8], which are briefly summarised here. The anti-$k_{t}$ $R=0.4$ algorithm was first run in four-momentum recombination mode on calorimeter cells grouped into $\Delta \eta \times \Delta \phi = 0.1\times 0.1$ calorimeter towers. The tower kinematics were obtained by summing electromagnetic-scale energies [30] of massless calorimeter cells within the tower boundaries. In the case of the reconstruction of jets in Pb+Pb collisions, an underlying event (UE) subtraction was performed in the following way. An iterative procedure was used to estimate a layer-dependent and pseudorapidity-dependent UE energy density while excluding jets from that estimate. The UE energy was corrected for the presence of the elliptic flow [31], which was subtracted from each calorimeter cell within the towers included in the reconstructed jet. The final jet kinematics were calculated via a four-momentum sum of all cell energy deposits (assumed massless) contained within the jet. The UE contribution was subtracted at the cell level. A correction was applied to the reconstructed jet to account for jets not excluded or only partially excluded from the UE estimate. Finally, the jet *y*- and $E_{\text {T}}$-dependent hadronic energy-scale calibration factor was applied in both the *pp* and Pb+Pb collisions.

In the trigger the HLT reconstruction algorithms described in Ref. [23] were used. The HLT jet trigger selection is fully efficient at a $p_{\mathrm{T}}$ of approximately 90 $\text {GeV}$. This, together with the intention to provide the results in the jet $p_{\mathrm{T}}$ selections that are the same as bins used in Ref. [10], limits the results to jets with $p_{\mathrm{T}}> 100~\text {GeV}$. The jet reconstruction performance is described in Ref. [8]. In order to evaluate the rapidity dependence of the jet structure, jets were categorised in four rapidity intervals, namely $|y|<0.3, 0.3<|y|<0.8, 1.2<|y|<2.1$, and $|y|<2.1$. The rapidity interval of $0.8<|y|<1.2$ was not considered in the analysis since the jet shape measurements are degraded in this region due to the transition in the detector between the SCT barrel and end-caps.

The tracks from *pp* collisions were required to have at least one hit in the pixel detector and six hits in the silicon microstrip detector. In order to reject secondary particles, the transverse ($d_0$) and longitudinal ($z_0\sin {\theta }$) impact parameters of the tracks measured with respect to the primary vertex were required to be smaller than 1.5 mm (0.2 mm for $d_0$ if $p_{\mathrm{T}}> 10~\text {GeV}$).

In Pb+Pb collisions, the occupancies of the three tracking subsystems reached different levels. The pixel detector occupancy was below 1% even in the most central collisions. The corresponding number for the SCT detector was below 10%, while the occupancy in the TRT reached 90% [32]. To account for the high occupancy in Pb+Pb events, the track reconstruction was configured differently from that in *pp* collisions. Tracks from Pb+Pb collisions were required to have at least two hits in the pixel detector, including a hit in the first pixel layer if the hit was expected from the track trajectory, and seven hits in the silicon microstrip detector. In addition, the $d_0$ and $z_0\sin {\theta }$ of the tracks measured with respect to the primary vertex were required to satisfy $|d_0/\sigma _{d_0} | < 3$ and $|z_0\sin {\theta }/\sigma _{z}| <3$, where $\sigma _{d_0}$ and $\sigma _{z}$ are uncertainties on $d_0$ and $z_0\sin {\theta }$, respectively, obtained from the track-fit covariance matrix. All tracks used in this analysis were required to have $p_{\mathrm{T}}> 1~\text {GeV}$.

The efficiency for reconstructing charged particles within jets was evaluated separately for *pp* and Pb+Pb collisions using MC events, described in Sect. 3. The efficiency was evaluated for charged particles that satisfy the selection criteria described above and were matched to generator-level (“truth”) jets with $p_{\mathrm{T}}> 100~\text {GeV}$ in each of the four jet rapidity intervals. In the case of Pb+Pb collisions, the efficiency was evaluated separately for each centrality bin.

**Fig. 1:**
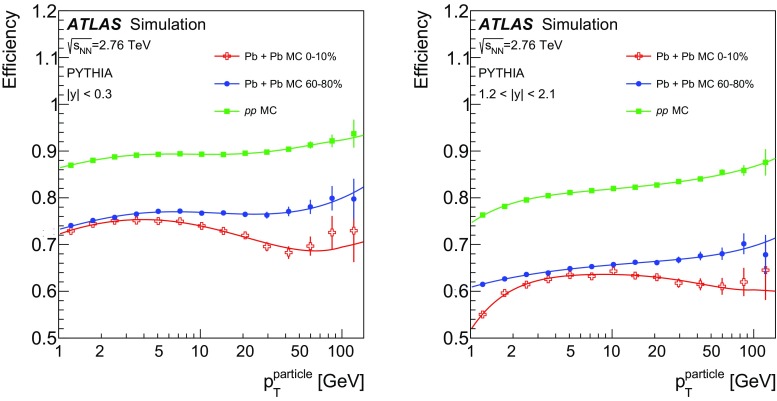
The tracking efficiency evaluated in simulation for particles in jets with $p_{\mathrm{T}}^{\mathrm{jet}}> 100~\text {GeV}$ as a function of truth charged-particle transverse momentum, $p_{\mathrm{T}}^{\mathrm{particle}}$, for jets with $|y| < 0.3$ (*left*) and $1.2< |y| < 2.1$ (*right*). Efficiency is shown for central and peripheral Pb+Pb collisions as well as for *pp* collisions. The *full line* represents the parameterisation (for more details see the body of the text)

The tracking efficiency correction $1/\varepsilon$ was evaluated as a function of charged-particle $p_{\mathrm{T}}$ and *y*. The tracking efficiency $\varepsilon$ was obtained as a ratio of tracks that have an associated truth charged particle to all the truth charged particles. To guarantee smooth behaviour of the correction factors as a function of track $p_{\mathrm{T}}$, the tracking efficiency was parameterised in the region of $1< p_{\mathrm{T}}< 90~\text {GeV}$ using a fourth-order polynomial in the logarithm of the track $p_{\mathrm{T}}$. This functional form gives a good description of the onset of the efficiency at low $p_{\mathrm{T}}$ as well as the behaviour in the intermediate-$p_{\mathrm{T}}$ region. At the same time it is not susceptible to statistical fluctuations in these regions. However, in the region of $p_{\mathrm{T}}>90~\text {GeV}$ the polynomial in the logarithm does not provide a good parameterisation of efficiencies. The study of the high-$p_{\mathrm{T}}$ behaviour in both the *pp* and Pb+Pb simulations showed that the tracking efficiency generally continues to follow the linear trend present at $p_{\mathrm{T}}\ < 90~\text {GeV}$. Thus, the result of the fit using a polynomial in the logarithm for tracks with $p_{\mathrm{T}}>90~\text {GeV}$ was replaced by a linear function with the slope determined from the difference between the fitted efficiencies at $p_{\mathrm{T}}=70~\text {GeV}$ and $p_{\mathrm{T}}=90~\text {GeV}$. The value of the slope does not exceed 0.001. The efficiency for reconstructing tracks along with its parameterisation is shown in Fig. 1. The fake-track contribution was evaluated by matching reconstructed tracks to truth MC particles and found to be smaller than 2% for tracks satisfying the selection requirements defined above.

## Analysis procedure

The analysis procedure is described briefly as follows. First, the measured distributions were corrected for the presence of a UE contribution (in the case of Pb+Pb collisions only) and for fake tracks. The corrected distributions were then unfolded using a two-dimensional Bayesian unfolding to correct for finite jet energy resolution and smearing due to finite track momentum resolution. The unfolded distributions were then normalised by the respective number of jets, which was obtained using one-dimensional Bayesian unfolding of jet $p_{\mathrm{T}}$ spectra. Details of each step in this procedure are discussed in the next paragraphs.

The first step in the analysis was to obtain measured two-dimensional uncorrected fragmentation functions, $D^{\mathrm{meas}}(z, p_{{\mathrm{T}}}^{{\mathrm{jet}}})$, and the two-dimensional distribution of charged-particle transverse momenta measured inside the jet, $D^{\mathrm{meas}}(p_{{\mathrm{T}}}^{\mathrm{ch}}, p_{{\mathrm{T}}}^{{\mathrm{jet}}})$, which are defined using the following formulae:

4 $$\begin{aligned} D^{\mathrm{meas}}(p_{{\mathrm{T}}}^{\mathrm{ch}}, p_{{\mathrm{T}}}^{{\mathrm{jet}}})\equiv & {} \frac{1}{\varepsilon (p_{{\mathrm{T}}}^{\mathrm{ch}}, y)} \frac{\Delta N_{\mathrm{ch}}(p_{{\mathrm{T}}}^{\mathrm{ch}}, p_{{\mathrm{T}}}^{{\mathrm{jet}}})}{\Delta p_{{\mathrm{T}}}^{\mathrm{ch}}}, \end{aligned}$$

5 $$\begin{aligned} D^{\mathrm{meas}}(z, p_{{\mathrm{T}}}^{{\mathrm{jet}}})\equiv & {} \frac{1}{\varepsilon (p_{{\mathrm{T}}}^{\mathrm{ch}}, y)} \frac{\Delta N_{\mathrm{ch}}(z, p_{{\mathrm{T}}}^{{\mathrm{jet}}})}{\Delta z}. \end{aligned}$$

Here $\Delta N_{\mathrm{ch}}(p_{{\mathrm{T}}}^{\mathrm{ch}})$ and $\Delta N_{\mathrm{ch}}(z)$ represent the number of measured charged particles within $\Delta R = 0.4$ of the jet axis obtained from the anti-$k_{t}$ clustering in given bins of charged-particle transverse momentum, $p_{{\mathrm{T}}}^{\mathrm{ch}}$, and *z* respectively.^5^ The variable $\varepsilon$ is the MC-evaluated track reconstruction efficiency. The superscript ‘meas’ in Eqs. (4) and (5) indicates that the measured distributions were corrected only for the tracking efficiency. The corrections for the UE and detector effects were applied in the subsequent steps of the analysis as discussed in the next paragraphs.

Charged particles from the UE constitute a background that needs to be subtracted from the measured distributions. This background depends on $p_{{\mathrm{T}}}^{\mathrm{ch}}$ and $\eta ^{\mathrm {ch}}$ of the charged particle, and the centrality of the collision. The contribution of the UE background was evaluated for each measured jet using a grid of $\Delta R = 0.4$ cones that spanned the full coverage of the inner detector. The cones had a fixed distance between their centres chosen such that the coverage of the inner detector was maximised while the cones did not overlap each other. To avoid biasing the UE estimate, cones associated with real jets have to be removed. This was done by removing cones having a charged particle with $p_{{\mathrm{T}}}^{\mathrm{ch}}>6$ $\text {GeV}$ or having a distance $\Delta R < 0.4$ between its centre and the nearest jet with $p_{\mathrm{T}}>90$ $\text {GeV}$.

The resulting UE charged-particle yields, $\mathrm{d}n_{\mathrm{ch}}^{\mathrm{UE}}/ \mathrm{d}p_{{\mathrm{T}}}^{\mathrm{ch}}$ or $\mathrm{d}n_{\mathrm{ch}}^{\mathrm{UE}}/ \mathrm{d}z$, were evaluated over $1< p_{{\mathrm{T}}}^{\mathrm{ch}}< 6$ $\text {GeV}$ as a function of charged-particle $p_{{\mathrm{T}}}^{\mathrm{ch}}$, $p_{{\mathrm{T}}}^{{\mathrm{jet}}}$, and $\eta ^{\mathrm{jet}}$ and averaged over all cones according to:

6 $$\begin{aligned} \dfrac{\mathrm{d}n_{\mathrm{ch}}^{\mathrm{UE}}}{ \mathrm{d}p_{{\mathrm{T}}}^{\mathrm{ch}}}= & {} \dfrac{1}{N_{\mathrm{cone}}} \dfrac{1}{\varepsilon } \dfrac{\Delta N_{\mathrm {ch}}^{\mathrm {cone}}(p_{{\mathrm{T}}}^{\mathrm{ch}},p_{{\mathrm{T}}}^{{\mathrm{jet}}}, \eta ^{\mathrm{jet}})}{\Delta p_{{\mathrm{T}}}^{\mathrm{ch}}}, \end{aligned}$$

7 $$\begin{aligned} \dfrac{\mathrm{d}n_{\mathrm{ch}}^{\mathrm{UE}}}{\mathrm{d}z}= & {} \dfrac{1}{N_{\mathrm{cone}}} \dfrac{1}{\varepsilon } \dfrac{\Delta N_{\mathrm {ch}}^{\mathrm {cone}}(z,p_{{\mathrm{T}}}^{{\mathrm{jet}}}, \eta ^{\mathrm{jet}})}{\Delta z} \Bigg |_{z = \frac{ p_\mathrm {T}^\mathrm {ch}}{ p_\mathrm {T}^\mathrm {jet}} \cos \Delta R }. \end{aligned}$$

Here $N_{\mathrm{cone}}$ represents the number of background cones associated with a given jet with $p_{{\mathrm{T}}}^{{\mathrm{jet}}}$ and $\eta ^{\mathrm{jet}}$, $\Delta N_{\mathrm {ch}}^{\mathrm {cone}}$ is the number of charged particles summed over all cones associated with the jet in question, and $\Delta R$ represents the distance between the centre of a cone and the direction of a given charged particle. Not shown in Eqs. (6) and (7) are correction factors that were applied to each background cone to correct for the difference in the average UE particle yield at a given $p_{{\mathrm{T}}}^{\mathrm{ch}}$ between the $\eta$ position of the cone and $\eta ^{\mathrm{jet}}$ and separate correction factors to account for the difference in the elliptic flow modulation at the $\phi$ position of the UE cone and $\phi ^{\mathrm{jet}}$. The former correction was based on a parameterisation of the $p_{{\mathrm{T}}}^{\mathrm{ch}}$ and centrality dependence of charged-particle yields in minimum-bias collisions. The latter correction was based on a parameterisation of the $p_{{\mathrm{T}}}^{\mathrm{ch}}$ and centrality dependence of elliptic flow coefficients, $v_2$, measured by ATLAS [22]. Since the measurement was not performed with respect to the reaction plane, the impact of the flow correction was at the level of a few percent of the UE yields. By evaluating the UE yields only from events containing jets included in the analysis, the background automatically had the correct distribution of centralities within a given centrality bin.

The UE yields need to be further corrected for the correlation between the actual UE yield in the jet and a finite, centrality-dependent jet energy resolution. Due to the steeply falling $p_{\mathrm{T}}$ distribution of jets, the smearing due to jet energy resolution leads to a net migration of jets from lower $p_{\mathrm{T}}$ to higher $p_{\mathrm{T}}$ values (hereafter referred to as “upfeeding”) such that a jet reconstructed with a given $p_{{\mathrm{T}}}^{\mathrm{rec}}$ corresponds, on average, to a truth jet with lower transverse momentum, $p_{\mathrm{T}}^{\mathrm{truth}}$. The upfeeding was observed in the MC simulation to induce a difference between the determined UE yields, as described above, and the actual UE contribution to reconstructed jets. This difference was found to be centrality dependent, and it also exhibited a weak $p_{{\mathrm{T}}}^{{\mathrm{jet}}}$ dependence. That difference was found to result from intrinsic correlations between the UE contribution to the yield of particles measured inside the jet and the MC $p_{{\mathrm{T}}}^{{\mathrm{jet}}}$ shift, $\Delta p_{\mathrm {T}}^{\mathrm {jet}} = p_{{\mathrm{T}}}^{\mathrm{rec}}- p_{\mathrm{T}}^{\mathrm{truth}}$. In particular, jets with positive (negative) $\Delta p_{\mathrm {T}}^{\mathrm {jet}}$ were found to have an UE contribution larger (smaller) than jets with $\Delta p_{\mathrm {T}}^{\mathrm {jet}} \sim 0$. Due to the net upfeeding in the falling jet spectrum, the selection of jets above a given $p_{{\mathrm{T}}}^{{\mathrm{jet}}}$ threshold causes the UE contribution to be larger than that estimated from the procedure described above. The average fractional mismatch in the estimated UE background was found to have a minor dependence on $p_{{\mathrm{T}}}^{\mathrm{ch}}$ and $p_{{\mathrm{T}}}^{{\mathrm{jet}}}$ and to vary with centrality by factors of 0–20% with respect to the original UE estimates. To correct for this effect, multiplicative correction factors, dependent on centrality, $y^\mathrm {jet}$, $p_{{\mathrm{T}}}^{{\mathrm{jet}}}$ and $p_{{\mathrm{T}}}^{\mathrm{ch}}$ (or *z*) were applied to the $\mathrm{d}n_{\mathrm{ch}}^{\mathrm{UE}}/ \mathrm{d}p_{{\mathrm{T}}}^{\mathrm{ch}}$ (or $\mathrm{d}n_{\mathrm{ch}}^{\mathrm{UE}}/ \mathrm{d}z$) distributions. These multiplicative factors were estimated in MC samples as a ratio of UE distributions calculated from tracks within the area of a jet which do not have an associated truth particle and the UE distributions estimated by the cone method. The measured distributions were also corrected for the presence of fake tracks by subtracting the fake-track contribution estimated in MC simulations. The corrected UE distributions, $\mathrm{d}\tilde{n}_{\mathrm{ch}}^{\mathrm{UE+fake}}/ \mathrm{d}p_{{\mathrm{T}}}^{\mathrm{ch}}$ and $\mathrm{d}\tilde{n}_{\mathrm{ch}}^{\mathrm{UE+fake}}/ \mathrm{d}z$, were then subtracted from measured distributions as follows:

8 $$\begin{aligned} D^{\mathrm{sub}}(p_{{\mathrm{T}}}^{\mathrm{ch}}, p_{{\mathrm{T}}}^{{\mathrm{jet}}})= & {} D^{\mathrm{meas}}(p_{{\mathrm{T}}}^{\mathrm{ch}}, p_{{\mathrm{T}}}^{{\mathrm{jet}}})- \frac{\mathrm{d}\tilde{n}_{\mathrm{ch}}^{\mathrm{UE+fake}}}{\mathrm{d}p_{{\mathrm{T}}}^{\mathrm{ch}}}, \end{aligned}$$

9 $$\begin{aligned} D^{\mathrm{sub}}(z, p_{{\mathrm{T}}}^{{\mathrm{jet}}})= & {} D^{\mathrm{meas}}(z, p_{{\mathrm{T}}}^{{\mathrm{jet}}})- \frac{ \mathrm{d}\tilde{n}_{\mathrm{ch}}^{\mathrm{UE+fake}}}{\mathrm{d}z}. \end{aligned}$$

While the correction for the UE can be large – in the most central collisions the UE exceeds the signal by more than a factor of ten – the correction for the presence of fake tracks is small, typically below 2%.

The UE and fake-track-subtracted measured distributions, $D^{\mathrm{sub}}(p_{{\mathrm{T}}}^{\mathrm{ch}}, p_{{\mathrm{T}}}^{{\mathrm{jet}}})$ and $D^{\mathrm{sub}}(z, p_{{\mathrm{T}}}^{{\mathrm{jet}}})$, need to be corrected for resolution effects. There are two main resolution effects: smearing due to finite jet energy resolution and smearing due to finite track momentum resolution. The former involves unfolding in $p_{{\mathrm{T}}}^{{\mathrm{jet}}}$; the latter involves unfolding in $p_{{\mathrm{T}}}^{\mathrm{ch}}$. Since the tracks were measured in jets, a two-dimensional unfolding needs to be used to correct for both of these resolution effects simultaneously. The two-dimensional Bayesian unfolding algorithm [33] from the RooUnfold package [34] was used for this purpose. Using the MC samples, four-dimensional response matrices were created using the truth and reconstructed $p_{{\mathrm{T}}}^{{\mathrm{jet}}}$ and the truth and reconstructed $p_{{\mathrm{T}}}^{\mathrm{ch}}$ for reconstructed charged particles satisfying the track selection criteria defined in Sect. 4. The response matrices were created separately for *pp* and Pb+Pb data for each centrality and rapidity range. The entries in the response matrix were weighted by the tracking efficiency correction. Five iterations in the Bayesian unfolding procedure were found sufficient to deliver a stable result that does not change with increasing numbers of iterations for all centrality bins except for the 0–10% centrality bin where, eight iterations were used. Once the two-dimensional distributions were unfolded, a projection to a given $p_{{\mathrm{T}}}^{{\mathrm{jet}}}$ interval was made, and the distribution was normalised by the respective number of jets.

The fragmentation distributions were measured for all jets reconstructed in the calorimeter, including those jets that do not contain any charged particle with $p_{{\mathrm{T}}}^{\mathrm{ch}}> 1~\text {GeV}$. The proper normalisation of the measured distributions by the number of jets requires a separate unfolding of the jet $p_{\mathrm{T}}$ spectrum. This was performed by applying a one-dimensional Bayesian unfolding, separately in each centrality and rapidity interval. One or two iterations were found to be sufficient for unfolding jet spectra in various centrality and rapidity intervals. The unfolded jet $p_{\mathrm{T}}$ spectra were integrated over a given jet $p_{\mathrm{T}}$ interval. The result of this integration represents the total number of jets spanning a given $p_{\mathrm{T}}$ interval and was used to normalise the unfolded fragmentation distributions, $D^{\mathrm{unfolded}}(p_{\text {T}})$ and $D^{\mathrm{unfolded}}(z)$, as follows

10 $$\begin{aligned} D(p_{\mathrm{T}})= & {} \frac{1}{N_\mathrm {jet}} D^{\mathrm{unfolded}}(p_{\text {T}}), \end{aligned}$$

11 $$\begin{aligned} D(z)= & {} \frac{1}{N_\mathrm {jet}} D^{\mathrm{unfolded}}(z), \end{aligned}$$

where $D(p_{\mathrm{T}})$ and *D*(*z*) are the final, particle-level corrected distributions that are presented in Sect. 7.

The performance of the reconstruction procedure was tested in MC samples by comparing unfolded distributions to truth distributions. Statistically independent MC samples for the response and reconstructed distributions were used. The ratio of unfolded to truth distributions was found to be consistent with unity for all the bins used in the measurement. An independent check of the subtraction of the UE contribution from measured distributions was performed by estimating the UE charged-particle $p_{\mathrm{T}}$ spectra from the minimum-bias data sample. After applying centrality reweighting, these UE charged-particle $p_{\mathrm{T}}$ spectra were found to be consistent within statistical uncertainties with UE distributions obtained by the cone method. The performance of the unfolding procedure was further tested in the data by a procedure in which unfolded distributions were folded back using the response matrix. These “refolded” distributions were then compared to original raw distributions. Only differences at sub-percent level between the raw distributions and the refolded distributions were found.

## Systematic uncertainties

The following sources of systematic uncertainty were identified for this measurement: the uncertainties in the jet energy scale (JES) and jet energy resolution (JER), the track reconstruction efficiency, and the unfolding. The systematic uncertainties were evaluated separately for distributions and their ratios for each rapidity and centrality selection.

The systematic uncertainty due to the JES has two contributions: the *pp* JES uncertainty and the heavy-ion JES uncertainty. The impact of the JES uncertainty on the measured distributions was determined by shifting the transverse momentum of reconstructed jets as follows:

12 $$\begin{aligned} p_{\mathrm{T}}' = p_{\mathrm{T}}\cdot (1 \pm U^\mathrm {JES}(p_{\mathrm{T}},y)), \end{aligned}$$

where $U^\mathrm {JES}(p_{\mathrm{T}},y)$ is either the *pp* JES uncertainty [30] or centrality-dependent heavy-ion JES uncertainty [35]. The distributions with shifted $p_{\mathrm{T}}$ were unfolded and compared to the original distributions. The fractional difference was used as an estimate of the systematic uncertainty. The size of the JES uncertainty for $D(p_{\mathrm{T}})$ and *D*(*z*) distributions in *pp* collisions is typically below 2% but can reach 4 and 6% at high $p_{\mathrm{T}}$ and *z*, respectively. In Pb+Pb collisions, the typical size of this uncertainty is the same as in *pp* collisions, but the maximal uncertainty can reach 15% at the largest $p_{\mathrm{T}}$ or *z*. The JES uncertainty partially cancels in ratios of Pb+Pb and *pp* distributions where a typical JES uncertainty is below 1% and the maximal uncertainty is below 10% at high $p_{\mathrm{T}}$. To account for systematic uncertainties due to any disagreement between the JER in data and MC simulation, the unfolding procedure was repeated with a modified response matrix. The new matrix was generated by repeating the MC study with the $p_{\mathrm{T}}$ of reconstructed jets smeared by a relative uncertainty estimated as a function of *y* and $p_{\mathrm{T}}$ of the jet [30]. The size of the JER uncertainty is usually at the level of 1% but grows at high $p_{\mathrm{T}}$ or *z*, where the maximum is $\approx$ $6\%$.

The systematic uncertainty due to track reconstruction was estimated by performing the analysis with three different sets of selection criteria imposed on tracks, called “loose”, “standard”, and “tight”. The standard selection criteria were used as a default in this analysis. The differences in the result obtained using loose and tight criteria with respect to the result obtained using the standard criteria were used as the estimate of the systematic uncertainty. The tight selection criteria imposed more stringent requirements on the track quality, leading to a 15–20% reduction of the tracking efficiency depending on the track $p_{\mathrm{T}}$, $\eta$, and centrality. The loose selection criteria imposed more relaxed requirements on track quality leading to a 5–10% enhancement of tracking efficiency. The differences in the selection criteria bring significant differences both in the magnitude and the $p_{\mathrm{T}}$dependence of the tracking efficiency. The track reconstruction uncertainty is usually largest systematic uncertainty at low and intermediate $p_{\mathrm{T}}$ or *z*. This uncertainty is typically less than 4%. Also related to tracking are the uncertainty in the estimate of fake tracks and the uncertainty due to the parameterisation of tracking efficiencies. Both of these uncertainties are less than 2%.

The unfolding procedure is sensitive to the MC model and the number of iterations used, $N_\mathrm {it}$. Two variations were implemented to account for this systematic uncertainty. First, the $N_\mathrm {it}$ was varied by $\pm 1$. Second, the MC response matrix was reweighted such that its projection onto the reconstructed axis matches the data. The data were then unfolded using the modified response matrix. The differences with respect to the original unfolded data were taken as the systematic uncertainty. The uncertainty due to unfolding was usually negligible and typically does not exceed 1%. To determine the total systematic uncertainty, the systematic uncertainties from all different sources were added in quadrature.

## Results

The measurements of the internal structure of jets were performed differentially in jet $p_{\mathrm{T}}$ and *y* and for two collision systems, *pp* and Pb+Pb. In the case of Pb+Pb collisions, the measurement was performed in seven bins of centrality, 0–10, 10–20, 20–30, 30–40, 40–50, 50–60, and 60–80%.

**Fig. 2:**
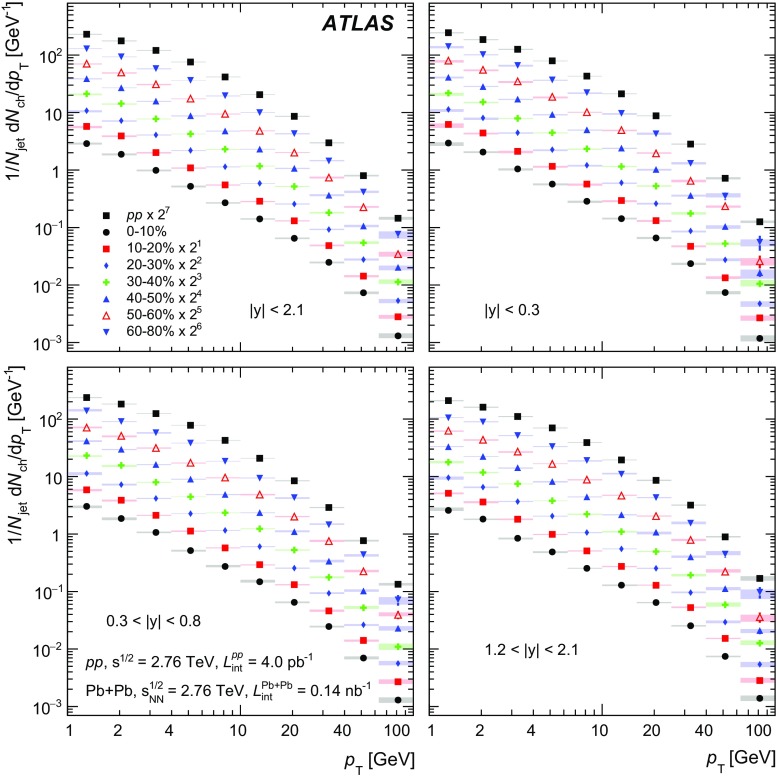
Unfolded charged-particle transverse momentum distributions, $D(p_{\mathrm{T}})$, measured in *pp* collisions and for seven centrality bins measured in Pb+Pb collisions. The *four panels* show $D(p_{\mathrm{T}})$ distributions with different selections in jet rapidity for jets with $p_{\mathrm{T}}$ in the interval of 100–398 $\text {GeV}$. The *error bars* on the data points indicate statistical uncertainties while the *shaded bands* indicate systematic uncertainties

**Fig. 3:**
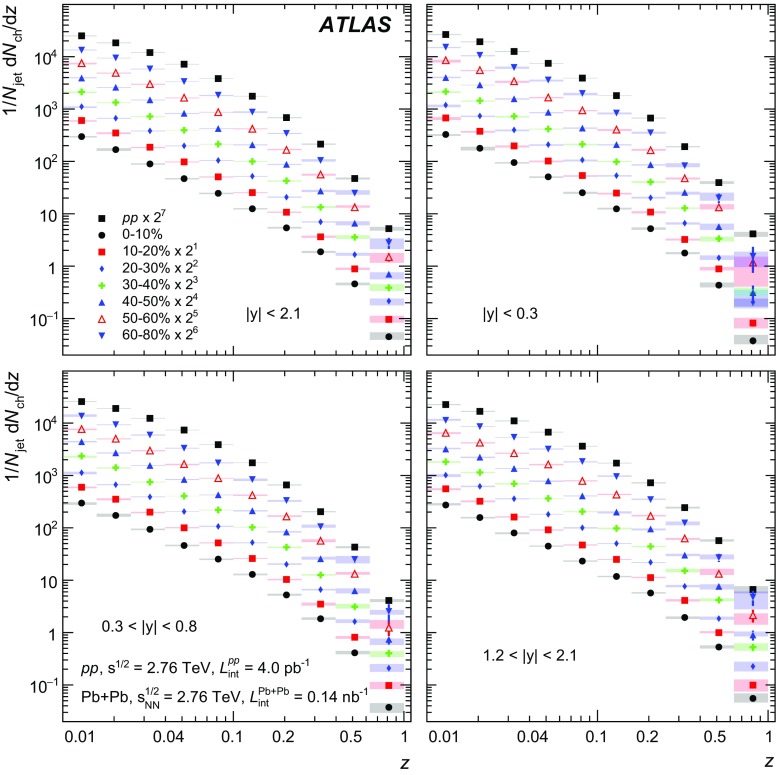
Unfolded distributions of longitudinal momentum fraction, *D*(*z*), measured in *pp* collisions and for seven centrality bins measured in Pb+Pb collisions. The four panels show *D*(*z*) distributions with different selections in jet rapidity for jets with $p_{\mathrm{T}}$ in the interval of 100–398 $\text {GeV}$. The error bars on the data points indicate statistical uncertainties while the shaded bands indicate systematic uncertainties

The measured distributions were evaluated in four different rapidity intervals of the jet: $|y|<2.1$, $|y|<0.3$, $0.3<|y|<0.8$, and $1.2<|y|<2.1$. The rapidity interval of $0.8<|y|<1.2$ was not considered in the analysis since the jet shape measurements are degraded in this region due to the transition in the detector between the SCT barrel and end-caps. This rapidity interval was also excluded from the measurement in the full rapidity range, $|y|<2.1$. The distributions were also evaluated in four different jet $p_{\mathrm{T}}$ intervals: $100< p_{{\mathrm{T}}}^{{\mathrm{jet}}}< 398~\text {GeV}$, $100< p_{{\mathrm{T}}}^{{\mathrm{jet}}}< 126~\text {GeV}$, $126< p_{{\mathrm{T}}}^{{\mathrm{jet}}}< 158~\text {GeV}$, and $158< p_{{\mathrm{T}}}^{{\mathrm{jet}}}< 398~\text {GeV}$. These intervals were chosen to correspond to intervals selected in the measurement of the jet nuclear modification factor [10]. This should allow the size of the energy lost by a jet, as quantified by the nuclear modification factor, to be connected to the respective modification of the jet fragmentation.

The $D(p_{\mathrm{T}})$ and *D*(*z*) distributions corrected to the hadron level by the unfolding procedure described in Sect. 5 are shown in Figs. 2 and 3, respectively. Different panels show distributions evaluated for different rapidity intervals for jets with $100< p_{\mathrm{T}}< 398~\text {GeV}$. The shaded band represents the total systematic uncertainty, while the error bars represent statistical uncertainties. The distributions exhibit a difference in shape between central heavy-ion collisions and peripheral heavy-ion collisions or the *pp* reference. To quantify this difference, the ratios of $D(p_{\mathrm{T}})$ and *D*(*z*) distributions measured in heavy-ion collisions to those measured in *pp* collisions were calculated and termed $R_{D(p_{\mathrm{T}})}$ and $R_{D(z)}$, respectively, following the nomenclature introduced in Ref. [13],

13 $$\begin{aligned} R_{D(p_{\mathrm{T}})} = D(p_{\mathrm{T}})|_\mathrm {cent} / D(p_{\mathrm{T}})|_{pp}, \quad R_{D(z)} = D(z)|_\mathrm {cent} / D(z)|_{pp}, \end{aligned}$$

where ‘cent’ represents one of the seven centrality bins.

**Fig. 4:**
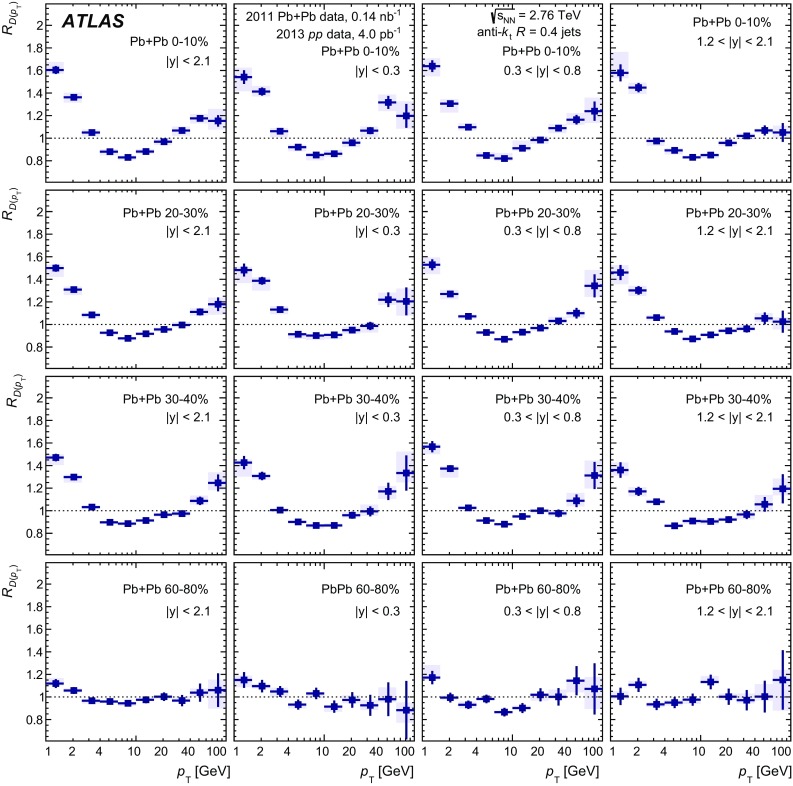
The ratio $R_{D(p_{\mathrm{T}})}$ of unfolded $D(p_{\mathrm{T}})$ distributions measured in heavy-ion collisions to unfolded $D(p_{\mathrm{T}})$ distributions measured in *pp* collisions. The $R_{D(p_{\mathrm{T}})}$ distributions were evaluated in four different centrality bins (*rows*) and four different selections in jet rapidity of jets (*columns*) with $100< p_{\mathrm{T}}< 398~\text {GeV}$. The *error bars* on the data points indicate statistical uncertainties while the *shaded bands* indicate systematic uncertainties

**Fig. 5:**
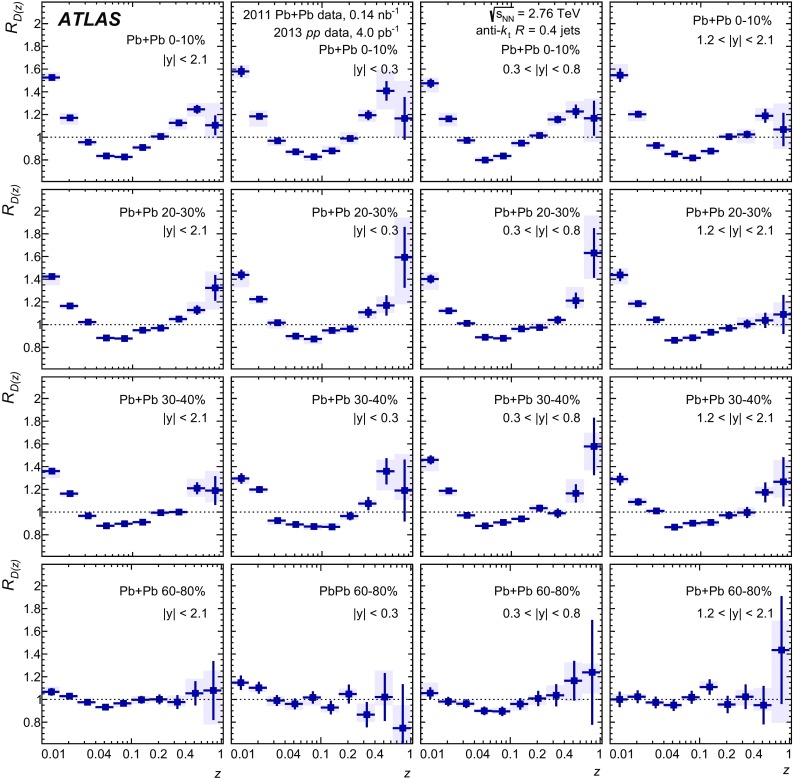
The ratio $R_{D(z)}$ of unfolded *D*(*z*) distributions measured in heavy-ion collisions to unfolded *D*(*z*) distributions measured in *pp* collisions. The $R_{D(z)}$ distributions were evaluated in four different centrality bins (*rows*) and four different selections in jet rapidity of jets (*columns*) with $100< p_{\mathrm{T}}< 398~\text {GeV}$. The *error bars* on the data points indicate statistical uncertainties while the *shaded bands* indicate systematic uncertainties

**Fig. 6:**
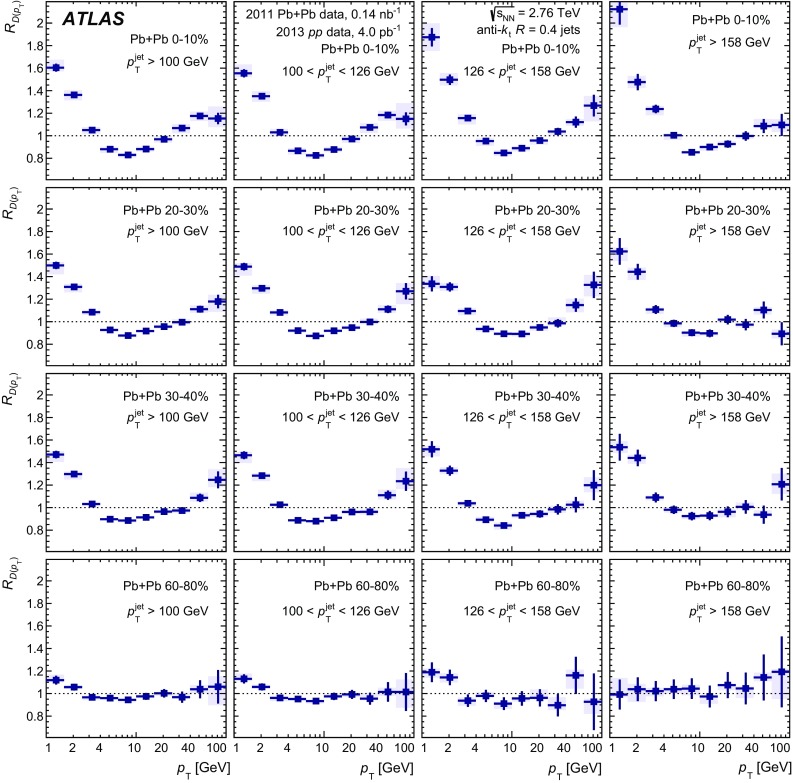
The ratio $R_{D(p_{\mathrm{T}})}$ of unfolded $D(p_{\mathrm{T}})$ distributions measured in heavy-ion collisions to unfolded $D(p_{\mathrm{T}})$ distributions measured in *pp* collisions. The $R_{D(p_{\mathrm{T}})}$ distributions were evaluated in four different centrality bins (*rows*) and four different selections in jet $p_{\mathrm{T}}$ of jets (*columns*) with $|y|<2.1$. The *error bars* on the data points indicate statistical uncertainties while the *shaded bands* indicate systematic uncertainties

**Fig. 7:**
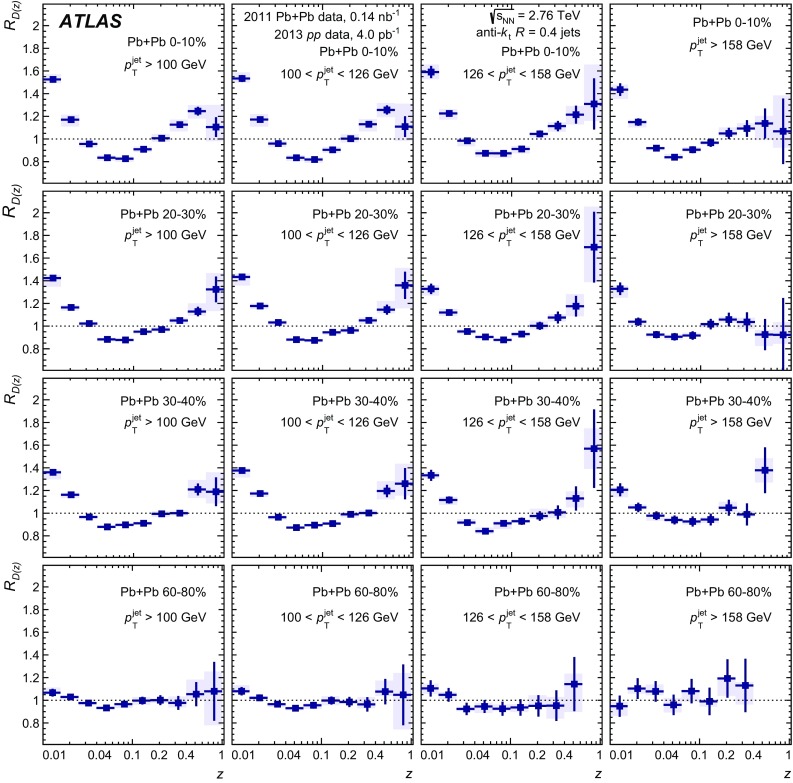
The ratio $R_{D(z)}$ of unfolded *D*(*z*) distributions measured in heavy-ion collisions to unfolded *D*(*z*) distributions measured in *pp* collisions. The $R_{D(z)}$ distributions were evaluated in four different centrality bins (*rows*) and four different selections in jet $p_{\mathrm{T}}$ of jets (*columns*) with $|y|<2.1$. The *error bars* on the data points indicate statistical uncertainties while the *shaded bands* indicate systematic uncertainties

The $R_{D(p_{\mathrm{T}})}$ and $R_{D(z)}$ distributions are shown in Figs. 4, 5, 6 and 7. Figure 4 shows the $R_{D(p_{\mathrm{T}})}$ distributions for four selections in collision centrality, namely 0–10, 20–30, 30–40 and 60–80%, and for four rapidity intervals of jets with $p_{{\mathrm{T}}}^{{\mathrm{jet}}}$ in the interval of 100–398 $\text {GeV}$. These ratios show an enhancement in fragment yield in central collisions for $p_{{\mathrm{T}}}^{\mathrm{ch}}<4~\text {GeV}$, a reduction in fragment yields for $4<p_{{\mathrm{T}}}^{\mathrm{ch}}<25~\text {GeV}$, and an enhancement in the fragment yield for $p_{{\mathrm{T}}}^{\mathrm{ch}}> 25~\text {GeV}$. The magnitude of these modifications decreases for more peripheral collisions. A similar observation is also made for the $R_{D(z)}$ distributions shown in Fig. 5. The characteristic shape of these ratios was also seen in the previous study [13] where the 60–80% bin was used as a reference. Figures 4 and 5 show that the difference in the modifications between different rapidity selections is marginal for fragments with $p_{{\mathrm{T}}}^{\mathrm{ch}}<25~\text {GeV}$ and $z<0.25$, respectively. Only at high $p_{{\mathrm{T}}}^{\mathrm{ch}}$ or high *z*, a small difference is observed – the enhancement is systematically lower for more forward jets than for jets measured in the central rapidity region.

Figures 6 and 7 show the $R_{D(p_{\mathrm{T}})}$ and $R_{D(z)}$ distributions, respectively, both for four $p_{{\mathrm{T}}}^{{\mathrm{jet}}}$ intervals of jets with $|y|<2.1$. No significant differences can be observed among the four $p_{{\mathrm{T}}}^{{\mathrm{jet}}}$ selections.

## Discussion

To quantify the trends seen in the ratios, the differences between integrals of $D(p_{\mathrm{T}})$ distributions measured in heavy-ion collisions and the integrals of $D(p_{\mathrm{T}})$ distributions measured in *pp* collisions, $N^\mathrm{ch}$, were evaluated,

14 $$\begin{aligned} N^\mathrm{ch}|_\mathrm {cent} \equiv \int _{p_{{\mathrm{T,min}}}}^{p_{{\mathrm{T,max}}}} (D(p_{\mathrm{T}})|_\mathrm {cent} - D(p_{\mathrm{T}})|_{{ pp}}) ~ \mathrm{d}p_{\mathrm{T}}. \end{aligned}$$

Three ranges defined by values of $p_{{\mathrm{T,min}}}$ and $p_{{\mathrm{T,max}}}$ were chosen to match the observations in $R_{D(p_{\mathrm{T}})}$, namely 1–4, 4–25, and 25–100 $\text {GeV}$. Thus three values of $N^\mathrm{ch}$ were obtained for each centrality bin which represent the number of particles carrying: (1) the excess seen in heavy-ion collisions for particles with $1< p_{\mathrm{T}}< 4~\text {GeV}$, (2) a depletion seen for particles with $4< p_{\mathrm{T}}< 25~\text {GeV}$, and (3) the enhancement seen for particles with $25< p_{\mathrm{T}}< 100~\text {GeV}$. Further, the differences between integrals of the first moment of the $D(p_{\mathrm{T}})$ distributions, $P_\mathrm{T}^\mathrm{ch}$, were also evaluated,

15 $$\begin{aligned} P_\mathrm{T}^\mathrm{ch}|_\mathrm {cent} \equiv \int _{p_{{\mathrm{T,min}}}}^{p_{{\mathrm{T,max}}}} ( D(p_{\mathrm{T}})|_\mathrm {cent} - D(p_{\mathrm{T}})|_{{ pp}} ) ~ p_{\mathrm{T}}~ \mathrm{d}p_{\mathrm{T}}. \end{aligned}$$

These differences represent the total transverse momentum of particles carrying the excess or the depletion observed in $R_{D(p_{\mathrm{T}})}$ distributions.

**Fig. 8:**
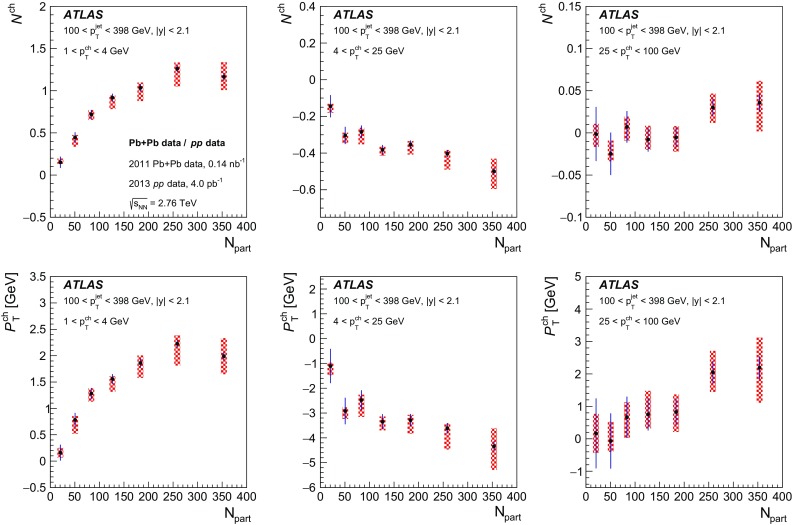
(*Upper panels*) The difference $N^\mathrm{ch}$ between the total yield of particles in a given $p_{{\mathrm{T}}}^{\mathrm{ch}}$ interval (indicated in the legend) measured in Pb+Pb collisions and the total yield of particles in the same $p_{{\mathrm{T}}}^{\mathrm{ch}}$ interval measured in *pp* collisions. (*Lower panels*) The difference $P_\mathrm{T}^\mathrm{ch}$ between the total transverse momentum of particles in a given $p_{{\mathrm{T}}}^{\mathrm{ch}}$ interval measured in Pb+Pb collisions and the total transverse momentum of particles measured in *pp* collisions. The differences were evaluated as a function of number of participating nucleons, $N_{\mathrm{part}}$. The *error bars* on the data points indicate statistical uncertainties while the *shaded bands* indicate systematic uncertainties

The result of performing this calculation is shown in Fig. 8 where the differences between the two integrals are displayed as a function of the number of participants, $N_{\mathrm{part}}$, calculated using the Glauber model analysis of the $\Sigma E_{\mathrm{T}}^{\mathrm{FCal}}$[22, 36, 37]. A clear, almost logarithmic, increase of yields of particles with low transverse momenta with increasing centrality is seen. In contrast, the intermediate-$p_{{\mathrm{T}}}^{\mathrm{ch}}$ region exhibits less significant modifications with varying centrality. The yield at high $p_{{\mathrm{T}}}^{\mathrm{ch}}$ shows a mild increase with increasing centrality, however with smaller significance. The changes in the total transverse momentum follow the trends seen in $R_{D(p_{\text {T}})}$ distributions. However, for the high-$p_{\mathrm{T}}$ region, the significance of the increase in yields is more pronounced in $R_{D(p_{\text {T}})}$ distributions than in the $P_\mathrm{T}^\mathrm{ch}$ distribution.

The difference defined in Eq. (15) can also be evaluated over the full range of charged-particle transverse momenta, $1< p_{{\mathrm{T}}}^{\mathrm{ch}}< 100~\text {GeV}$. It may be expected that such $P_\mathrm{T}^\mathrm{ch}$ should be identical to zero since the same range of the $p_{{\mathrm{T}}}^{{\mathrm{jet}}}$ was used in Pb+Pb and *pp* collisions. The result of this evaluation is presented in the second row of Table 1. Indeed, the $P_\mathrm{T}^\mathrm{ch}$ evaluated over the full range of charged-particle transverse momenta is consistent with zero within one standard deviation of combined statistical and systematic uncertainties. The small residual deviations from zero are likely due to the difference in the shape of $p_{{\mathrm{T}}}^{{\mathrm{jet}}}$ spectra between *pp* and Pb+Pb collisions [10], which leads to a difference in the mean $p_{{\mathrm{T}}}^{{\mathrm{jet}}}$ between Pb+Pb and *pp* collisions.

The total difference in the yield of charged particles can also be evaluated by integrating the $D(p_{\mathrm{T}})$ distributions over the full range of charged-particle transverse momenta. In this case, one does not expect to see the same yields of charged particles in Pb+Pb and *pp* collisions since this quantity may change as a result of the jet quenching. The resulting $N^\mathrm{ch}$ is summarised in the bottom row of Table 1.

**Table 1:** The difference between *pp* and Pb+Pb collisions in the total momentum, $P_\mathrm{T}^\mathrm{ch}$, and the total difference in the yield of charged particles between *pp* and Pb+Pb collisions, $N^\mathrm{ch}$, evaluated over the full range of charged-particle transverse momenta, $1< p_{{\mathrm{T}}}^{\mathrm{ch}}< 100~\text {GeV}$, and for different values of centrality

Centrality	0–10%	10–20%	20–30%	30–40%	40–50%	50–60%	60–80%
$$P_\mathrm{T}^\mathrm{ch}$$ ($$\text {GeV}$$)	$$ 0.9^{ + 0.9}_{ - 1.7}$$	$$ 1.0^{ + 0.8 }_{ -1.3}$$	$$ -0.0^{ + 0.7}_{ - 1.1}$$	$$ -0.6^{ + 0.8}_{ - 0.8} $$	$$ -0.5^{ + 1.0}_{ - 1.2} $$	$$ -1.4^{ + 1.0}_{ - 1.2} $$	$$ -0.8^{ + 1.3}_{ - 1.4} $$
$$N^\mathrm{ch}$$	$$ 0.7^{ + 0.1}_{ -0.2 }$$	$$ 0.9^{ + 0.1}_{ -0.1 } $$	$$ 0.7^{ + 0.1}_{ -0.1 } $$	$$ 0.5^{ + 0.1}_{ -0.2 }$$	$$ 0.4^{ + 0.1}_{ -0.1 }$$	$$ 0.2^{ + 0.1}_{ -0.2 } $$	$$ 0.0^{ + 0.1}_{ -0.1 }$$

**Fig. 9:**
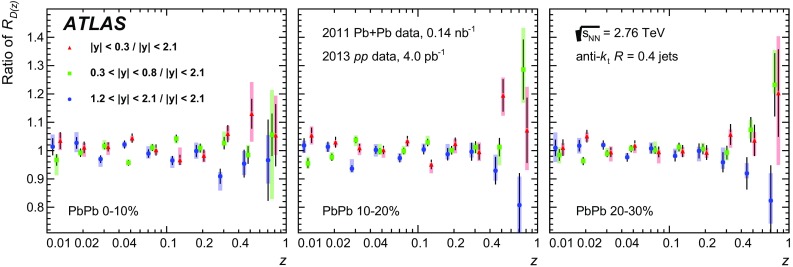
The ratio of $R_{D(z)}$ distributions in a given rapidity interval, namely $|y|<0.3$, $0.3<|y|<0.8$, and $1.2<|y|<2.1$, to $R_{D(z)}$ in $|y|<2.1$. The ratio of $R_{D(z)}$ was evaluated for three different collision centralities. The *error bars* on the data points indicate statistical uncertainties while the *shaded bands* indicate systematic uncertainties

The enhancement of fragment yields at low $p_{\mathrm{T}}$ or *z* already reported in previous analyses [13, 15] is confirmed, and it is consistent with a jet quenching interpretation in which the energy lost by partons is transferred predominantly to soft particles [17]. While the enhancement of soft fragments may be understood as a direct consequence of the parton energy loss, the enhancement of fragment yields at high $p_{\mathrm{T}}$ or *z* is unexpected [38]. A discussion of this feature in terms of different quenching of quark and gluon jets was recently provided in Ref. [39]. In order to further study this enhancement the ratio of $R_{D(z)}$ distributions in a given rapidity interval to $R_{D(z)}$ in $|y|<2.1$ is evaluated and plotted in Fig. 9. At high *z* ($z \gtrsim 0.4$) the result shows a trend of enhancements in the ratio of $R_{D(z)}$ measured in $|y|<0.3$ to $R_{D(z)}$ in $|y|<2.1$ and a trend of depletions in the ratio of $R_{D(z)}$ measured in $1.2<|y|<2.1$ to $R_{D(z)}$ in $|y|<2.1$.

## Summary

This paper presents a measurement of internal structure of jets performed with the ATLAS detector at the LHC. The distributions of charged-particle transverse momentum and longitudinal momentum fraction are measured in jets reconstructed using the anti-$k_{t}$ algorithm with $R=0.4$. These distributions are measured differentially in jet $p_{\mathrm{T}}$, jet rapidity, and in Pb+Pb as well as *pp* collisions at a centre-of-mass energy of 2.76 TeV per colliding nucleon pair. The Pb+Pb and *pp* data correspond to integrated luminosities of 0.14 nb$^{-1}$ and 4.0 pb$^{-1}$, respectively. In the case of Pb+Pb collisions, the measurements are performed in bins of collision centrality. The distributions measured in *pp* collisions are used as a reference for the distributions measured in Pb+Pb collisions to evaluate the impact of the jet energy loss on the internal structure of jets. The measurements cover the jet $p_{\mathrm{T}}$ range of 100–398 $\text {GeV}$ and use charged particles with $p_{\mathrm{T}}>1~\text {GeV}$. The results are corrected to the hadron level.

The ratios of charged-particle transverse momentum distributions measured in Pb+Pb collisions to those measured in *pp* exhibit an enhancement in fragment yield in central collisions for $1<p_{{\mathrm{T}}}^{\mathrm{ch}}<4~\text {GeV}$, a reduction in fragment yields for $4<p_{{\mathrm{T}}}^{\mathrm{ch}}<25~\text {GeV}$, and an enhancement in the fragment yield for $p_{{\mathrm{T}}}^{\mathrm{ch}}> 25~\text {GeV}$. The magnitude of these modifications decreases in more peripheral collisions. A similar observation is also made for the distributions of longitudinal momentum fraction measured with respect to the jet axis.

The centrality dependence of the magnitude of modifications was further quantified by evaluating the differences between integrals of charged-particle transverse momentum distributions measured in Pb+Pb and *pp* collisions for these three characteristic $p_{{\mathrm{T}}}^{\mathrm{ch}}$ intervals. Further, the jet $p_{\mathrm{T}}$- and *y*-dependence of the modifications in the internal structure of jets was measured. In addition, no significant differences in modifications of the jet structure are observed among different $p_{{\mathrm{T}}}^{{\mathrm{jet}}}$ selections spanning the interval of 100–398 $\text {GeV}$. No significant evolution in modifications of the jet structure as a function of rapidity are observed except for a difference at high $p_{{\mathrm{T}}}^{\mathrm{ch}}$ or high *z*, where a hint of reduction of the enhancement for more forward jets is observed.

These new results improve our understanding of the in-medium modifications of parton showers and help to constrain jet-quenching models.

## Footnotes

1 ATLAS uses a right-handed coordinate system with its origin at the nominal interaction point (IP) in the centre of the detector and the *z*-axis along the beam pipe. The *x*-axis points from the IP to the centre of the LHC ring, and the *y*-axis points upward. Cylindrical coordinates $(r,\phi )$ are used in the transverse plane, $\phi$ being the azimuthal angle around the beam pipe. The pseudorapidity is defined in terms of the polar angle $\theta$ as $\eta =-\ln \tan (\theta /2)$. Rapidity is defined as $y = 0.5 \ln \frac{E+p_z}{E-p_z}$ where *E* and $p_z$ are the energy and the component of the momentum along the beam direction.

2 The $\Delta R \equiv \sqrt{(\Delta \eta )^2 + (\Delta \phi )^2}$ used here is a boost-invariant replacement for the polar angle $\theta$ between the jet and charged particle.

3 Except the third sampling layer, which has a segmentation of $0.2 \times \pi /32$ up to $|\eta | = 1.7$.

4 While generator-level charged particles are massive, the tracks reconstructed in the inner detector are massless.

5 The labels ‘ch’ and ‘jet’ are used here to better distinguish the quantities connected with charged particles from quantities connected with jets.
